# Agro-Morphological and Molecular Characterization Reveal Deep Insights in Promising Genetic Diversity and Marker-Trait Associations in *Fagopyrum esculentum* and *Fagopyrum tataricum*

**DOI:** 10.3390/plants12183321

**Published:** 2023-09-20

**Authors:** Barbara Pipan, Lovro Sinkovič, Mohamed Neji, Dagmar Janovská, Meiliang Zhou, Vladimir Meglič

**Affiliations:** 1 Crop Science Department, Agricultural Institute of Slovenia, Hacquetocva ulica 17, SI-1000 Ljubljana, Slovenia; lovro.sinkovic@kis.si (L.S.); mohamed.neji@kis.si (M.N.); vladimir.meglic@kis.si (V.M.); 2 Gene Bank, Crop Research Institute, Drnovská 507, 161 06 Prague, Czech Republic; janovska@vurv.cz; 3 Institute of Crop Sciences, Chinese Academy of Agricultural Sciences, Room 420, National Crop Genebank Building, Zhongguancun South Street No. 12, Haidian District, Beijing 100081, China; zhoumeiliang@caas.cn

**Keywords:** agro-morphological traits, genetic diversity, microsatellite markers, buckwheat, marker-trait associations, breeding

## Abstract

Characterisation of genetic diversity is critical to adequately exploit the potential of germplasm collections and identify important traits for breeding programs and sustainable crop improvement. Here, we characterised the phenotypic and genetic diversity of a global collection of the two cultivated buckwheat species *Fagopyrum esculentum* and *Fagopyrum tataricum* (190 and 51 accessions, respectively) using 37 agro-morphological traits and 24 SSR markers. A wide range of variation was observed in both species for most of the traits analysed. The two species differed significantly in most traits, with traits related to seeds and flowering contributing most to differentiation. The accessions of each species were divided into three major phenoclusters with no clear geographic clustering. At the molecular level, the polymorphic SSR markers were highly informative, with an average polymorphic information content (PIC) of over 0.65 in both species. Genetic diversity, as determined by Nei’s expected heterozygosity (He), was high (He = 0.77 and He = 0.66, respectively) and differed significantly between species (*p* = 0.03) but was homogeneously distributed between regions, confirming the lack of genetic structure as determined by clustering approaches. The weak genetic structure revealed by the phenotypic and SSR data and the low fixation indices in both species suggested frequent seed exchange and extensive cultivation and selection. In addition, 93 and 140 significant (*p* < 0.05) marker-trait associations (MTAs) were identified in both species using a general linear model and a mixed linear model, most of which explained >20% of the phenotypic variation in associated traits. Core collections of 23 and 13 phenotypically and genetically diverse accessions, respectively, were developed for *F. esculentum* and *F. tataricum*. Overall, the data analysed provided deep insights into the agro-morphological and genetic diversity and genetic relationships among *F. esculentum* and *F. tataricum* accessions and pointed to future directions for genomics-based breeding programs and germplasm management.

## 1. Introduction

Buckwheat (*Fagopyrum* spp.) is a dicot pseudo-cereal belonging to the Polygonaceae family and native to China, where its domestication dates back ∼4000 years [1]. The genus *Fagopyrum* includes 20 species, of which *Fagopyrum esculentum* (common buckwheat) and *Fagopyrum tataricum* (Tartary buckwheat) are the most important species cultivated worldwide [2]. Although the two species are diploid (2n = 2x = 16) and characterised by a short growth span (∼70 days), *F. esculentum* is outcrossing and has a relatively larger genome (∼450 Mbp) compared to *F. tataricum*, a predominantly self-fertile species [3]. The genome of *F. tataricum* has been shown to contain duplication events that allow this species to better adapt to harsh environments [4]. Both species are nutritionally valuable food sources due to their content of proteins, lipids, fibre, vitamins, and minerals, and contain various health-promoting components such as phenolic acids, condensed tannins, flavonoids, and phytosterols, which endues these two gluten-free crops with great medicinal value [5,6,7]. However, due to their contrasting biological and ecological characteristics, the two species differ greatly in their cultivation areas around the world [8], with *F. esculentum* being widely cultivated on all continents, while *F. tataricum* cultivation is limited to China and India in Asia and to several areas in Europe [9,10,11]. Both species harbour great agro-morphological and genetic diversity that can be exploited for future agriculture [12]. However, despite their immense agricultural superiority, the lack of effective improvement measures compared to other crops, changing cropping patterns, and ongoing climate change have led to a noticeable decline in the global acreage of buckwheat species and an alarming depletion of their genetic diversity in recent years [13,14]. To date, about 10,000 buckwheat accessions are stored in various genebanks around the world dedicated to the conservation of buckwheat genetic resources [12]. However, a large number of buckwheat accessions have been phenotypically characterised based on agro-morphological traits, and detailed genetic characterisation makes it difficult to manage and use them for buckwheat improvement [15,16,17,18]. Other previous studies have used various molecular makers, including randomly amplified polymorphic DNA (RAPD) [19,20], amplified fragment-length polymorphisms (AFLPs) [21], inter simple sequence repeats (ISSRs) [22], and simple sequence repeats (SSRs) [23,24,25]. Most of these studies have provided insights into only a tiny fraction of the genetic diversity of common and Tartary buckwheat. In fact, hardly any studies included a global collection, and those that did include often relied on a small number of accessions or a small number of phenotypic traits or markers, which can lead to biased estimates of genetic diversity and weak inferences about genetic structure. Compared to other DNA-based markers, SSRs are best suited for characterising genetic diversity due to their high reproducibility, abundance, codominance, and multi-allelic inheritance [26]. In addition, Hou et al. [27] have demonstrated the great utility of combining SSRs and agro-morphological markers in identifying genomic regions controlling important agronomic traits in Tartary buckwheat.

In-depth characterisation of the agro-morphological and genetic diversity of diverse germplasm is indispensable for identifying genomic regions controlling important agronomic traits and elite accessions holding favourable alleles that can be used for future marker-assisted breeding programs. In this study, we performed a detailed phenotypic and molecular characterisation of a diverse worldwide collection of the two closely related buckwheat species, *F. esculentum* (common buckwheat) and *F. tataricum* (Tartary buckwheat), with contrasting mating systems using a large set of agro-morphological traits and SSR markers in order explore in details the genetic relationship between the two species and contribute to better understanding of the role of mating systems on the patterns of their genetic diversity and structure. Importantly, this comparative analysis will aid in the development of breeding programs that efficiently utilise the available germplasm of these two main cultivated buckwheat species. Hence, the major aims of this study were (i) to provide an in-depth characterisation of phenotypic and genetic diversity and structure and (ii) to identify marker-trait associations (MTAs) related to important agro-morphological traits. In addition, the data obtained were used to develop phenotypically and molecularly informed core collections for both species to facilitate future genetic studies and breeding programs for buckwheat. However, the importance of our study is also providing trait-focused information about the two most widely cultivated buckwheat species for breeders all over the world, to implement the most interesting genetic material into breeding programmes considering variable breeding objectives and finally to exploit the precious genetic potential of buckwheat germplasm.

## 2. Results

### 2.1. Patterns of Agro-Morphological Variation and Correlation between Traits

Substantial agro-morphological variability, expressed by the coefficient of variation (CV %), was observed between accessions in most quantitative traits of the two species studied, with CV averaging 15.17% and 15.58% for *F. esculentum* and *F. tataricum*, respectively. Similar patterns of variation were observed for most traits in both species. The highest variation was observed in the number of flower clusters per cyme, with a CV of 26.07% and 26.09% for *F. esculentum* and *F. tataricum*, respectively. The remaining traits showed moderate (10% ≤ CV ≤ 20%) variation in both species, except for days to flowering (CV = 7.26%) and seed length (CV = 6.63%) in *F. esculentum* and days to flowering (CV = 6.26%) in *F. tataricum* (Table 1). On the other hand, the Kruskal–Wallis test showed that the two species were significantly different for ten of the 12 quantitative traits (*p* < 0.0001). However, the ranges of values (minimum and maximum) overlapped for most traits (Table 1).

Similar to quantitative traits, overall phenotypic diversity expressed by the Shannon diversity index (*H*′) was similar in both species, with mean values of 0.77 and 0.75 for the 25 observed traits in *F. esculentum* and *F. tataricum*, respectively (Table S2). Noteworthy, all accessions of *F. esculentum* had green leaf margin colour, whereas the 51 accessions of *F. tataricum* were homogeneous with respect to floral morphology/self-pollinated nature (stamens as long as style), inflorescence compactness (cyme loose), and seed surface (irregular). Among the polymorphic traits, the highest degree of diversity was observed for leaf blade shape (*H*′ = 1.61) in *F. esculentum* and for leaf margin colour (*H*′ = 1.59) in *F. tataricum*, while seed surface and time to maturity were the least polymorphic in both species, with *H*′ values of 0.03 and 0.10, respectively (Table S2). In addition, Fisher’s exact test revealed that the two species differed significantly in 21 of the 25 traits analysed (Table S2). In general, *F. esculentum* had green leaf margin colour, branched inflorescence, white flower, and smooth and trullate seeds. *F. tataricum*, on the other hand, was mainly characterised by a loose inflorescence, stamens as long as style and irregular and brown seeds.

The associations between all pairs of traits of both species (Table S4) showed that 576 and 538 correlations were significant, of which 21 and 114 were moderate (0.4 ≤ |ρ| ≤ 0.59) to very strong (0.8 ≤ |ρ| ≤ 0.1) in *F. esculentum* and *F. tataricum*, respectively (*p* < 0.01). Although the direction of the relationship (positive or negative) was the same in both species, the strength was generally higher in *F. tataricum*. In both species, the strongest positive correlations were observed between cotyledon anthocyanin colouration and cotyledon/seedling leaf colour (>0.85) and between pairwise seed-related traits such as 1000-seed weight, seed width, seed length, and seed surface area (0.62–0.82), most likely reflecting the strong developmental relationships between these traits. In contrast, weak to very weak significant negative associations were observed between the pair formation of all the remaining traits in *F. esculentum*, except for the moderately negative correlation between the number of nodes per stem and plant height (ρ = −0.53). In *F. tataricum*, the most relevant negative associations were observed between growth and branching form and the number of nodes per stem with flower-related traits.

### 2.2. Patterns of Agro-Morphological Differentiation among and within Species

**Figure 1:**
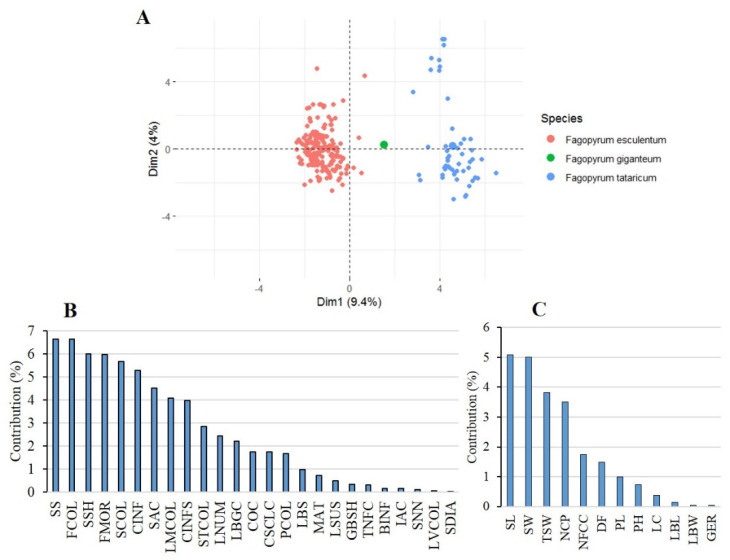
(**A**) Two-dimensional MFA plots showing the patterns of phenotypic differentiation between *F. esculentum* and *F. tataricum* accessions based on the data of the 37 agro-morphological traits. Contribution of the 25 quantitative (**B**) and 12 qualitative (**C**) agro-morphological traits to the total variance explained by the first three dimensions of the multifactorial analysis (MFA).

Multifactorial analysis showed that *F. esculentum* and *F. tataricum* were clearly distinguished from each other along the first dimension, which explained 9.4% of the total variance, based on the data from the 37 traits analysed (Figure 1A). The qualitative traits that contributed most to the first MFA dimension (Dim-1) were mainly seed surface, flower colour, seed shape, flower morphology, seed colour, and the colour of the inflorescence stalk, which contributed more than 5% to the total variance (Figure 1B). On the other hand, seed length, seed weight, 1000-seed weight, and number of cymes per plant contributed most to the quantitative variance of Dim-1 (Figure 1B). Thus, the agro-morphological differentiation between the two species was mainly related to seed- and flower-related traits.

**Figure 2:**
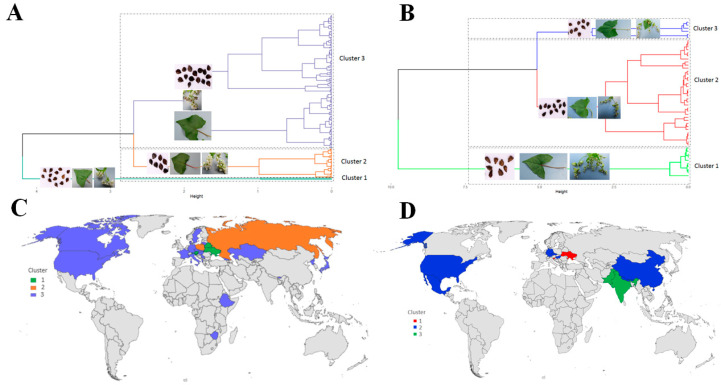
Hierarchical clustering on multifactorial analysis showing the number of phenotypic groups in *F. esculentum* (**A**) and *F. tataricum* (**B**). Geographical maps showing the distribution of the phenotypic groups of *F. esculentum* (**C**) and *F. tataricum* (**D**).

Furthermore, the accessions of each species were classified into homogeneous phenoclusters using the hierarchical clustering of MFA principal components (HCPC). The 190 accessions of *F. esculentum* were divided into three major phenoclusters. Most accessions (177) were grouped in a large phenocluster (cluster 3) characterised by a branched inflorescence with semi-compact cyme, a high number of flower clusters, a stem with many nodes, a long leaf blade and cyme, and tall plants (Figure 2A). The first cluster included 11 accessions mainly characterised by non-branching inflorescence, determinate growth habit, short plants, low number of flower clusters per cyme, and short cyme and plant length, while the second cluster included only two accessions characterised by pink petioles and flowers. On the other hand, the 51 accessions of *F. tataricum* were divided into three phenoclusters (Figure 2B). The first cluster included ten accessions very susceptible to plant lodging and characterised by semi-erect growth and branched shoots, unbranched inflorescences, a high number of cymes per plant, short plants, and ovoid seeds. The second cluster included 35 accessions characterised by elliptical seeds, a red inflorescence stalk, multi-coloured flowers (greenish-yellow/pink) with weak anthocyanin colouration, a high number of flower clusters per cyme, long cymes and seeds, and a large leaf blade. The third cluster included six accessions characterised by multi-coloured leaves (green/pink/red), a very tall plant, small seeds, and a low 1000-seed weight. The last cluster also included four accessions characterised by multi-coloured leaf margins (green/red), inflorescences (green/pink) and petioles (green/pink/red), late flowering, high number of flower clusters per cyme, and long cyme. Geographic space diagrams showed that *F. esculentum* accessions were not clustered by country of origin or geographic region (Figure 2C). However, some geographic clustering was observed in *F. tataricum*. Cluster 1 included only Slovenian accessions; the second cluster consisted of Asian accessions from Pakistan and Bhutan. In contrast, the third cluster consisted of a mixture of accessions from different countries, with most accessions coming from America (12) and Asia (13) (Figure 2D).

### 2.3. Microsatellite Polymorphism and Species-Level Diversity

The 24 SSR markers analysed in our study were transferable to common and Tartary buckwheat and were highly polymorphic in both species, except for the marker Fem1303, which was monomorphic in Tartary buckwheat. A total of 205 and 118 alleles were detected in *F. esculentum* and *F. tataricum*. Of all the alleles detected, 104 alleles were unique common buckwheat, only 18 alleles were unique Tartary buckwheat, while 100 alleles were shared between the two species.

In *F. esculentum*, the maximum number of alleles (Na = 25) and the highest allelic richness (Ar = 15.47) were observed in SXAU048, while the lowest Na and Ar values were observed in TBP_6 with Na and Ar mean values of 13,95 and 8.64, respectively, across all loci. Major allele frequency (MAF) varied from 0.18 in Fem1322 to 0.65 in Fem1303, with a mean of 0.35 (Table 2). With values ranging from 0.43 in TBP_6 to 0.89 in SXAU089 and a mean of 0.74, the polymorphic information content values (PIC) confirmed the high polymorphism of the 24 SSR markers. Both observed (Ho) and expected heterozygosity (He) varied widely among the markers, with Ho values ranging from 0.06 for TBP6 to 1 for GB_FE_035 and He values ranging from 0.51 for TBP6 and 0.90 for SXAU089. On average, He was slightly higher than Ho, with mean values of 0.82 and 0.77, respectively (Table 2). With values ranging from −0.75 (GB_FE_035) to 0.89 (TBP6) and 18 markers showing negative values, the average fixation index value for all 24 markers was slightly positive (Fis = −0.07), indicating a slight excess of heterozygosity. In *F. tataricum*, the highest Na, Ar, PIC, Ho, and He values, and the lowest MAF value were recorded in Ft5_2899 (Na = 14; Ar = 13.99, MAF = 0.2, PIC = 0.89, Ho = 0.96, and He = 0.89) and Ft3_572 (Na = 14; Ar = 13.92, MAF = 0.22, PIC = 0.88, Ho = 0.90, and He = 0.88), suggesting that these two loci were the most polymorphic. In contrast, the SXAU060 572 loci (Na = 2; Ar = 2, MAF = 0.63, PIC = 0.36, Ho = 0, and He = 0.47) appeared to be the least polymorphic. At all loci, except the monomorphic locus Fem1303, the average Na, Ar, MAF, PIC, Ho, and He values were 6.38, 6.34, 0.44, 0.61, 0.74, and 0.66, respectively. As with *F. esculentum*, most of the markers analysed showed a negative fixation index with values ranging from -0.85 at Ft2_1743 to 1 at SXAU060 and an average value of -0.12 across all 23 polymorphic loci.

As mentioned earlier, *F. esculentum* appears to be more genetically diverse than *F. tataricum*. This was confirmed by analysis of variance, which revealed that the two species differed significantly in allelic richness (Ar = 9.15; *p* = 0.029), observed number of alleles (Na = 28.02; *p* = 0.003), and expected heterozygosity (He = 8.42; *p* = 0.034). Although Africa and Eurasia were not represented in *F. tataricum*, non-significant differences were found among regions within the species for all indices of genetic diversity, indicating that there was little variation in genetic diversity among regions (Table 3).

Analysis of molecular variance (AMOVA) was conducted to estimate patterns of genetic variation at the species and accession levels. AMOVA revealed that although most of the genetic variation was explained by differences within accessions (78.09%), significantly high variation occurred between the two species (21.09%), while no variation was detected between accessions within species, indicating substantial genetic differentiation between the two species (Table 4). Further AMOVA analyses were conducted using region and country of origin as factors to examine whether either factor influenced patterns of genetic variation within each species. These analyses showed that in both species, most of the genetic variation was due to heterozygosity within accessions (97.79% in *F. esculentum* and 91.02% in *F. tataricum*) and that genetic variation was not significantly affected by region and country of origin of the accessions (Table 4).

### 2.4. Genetic Differentiation and Population Structure between and within Species

**Figure 3:**
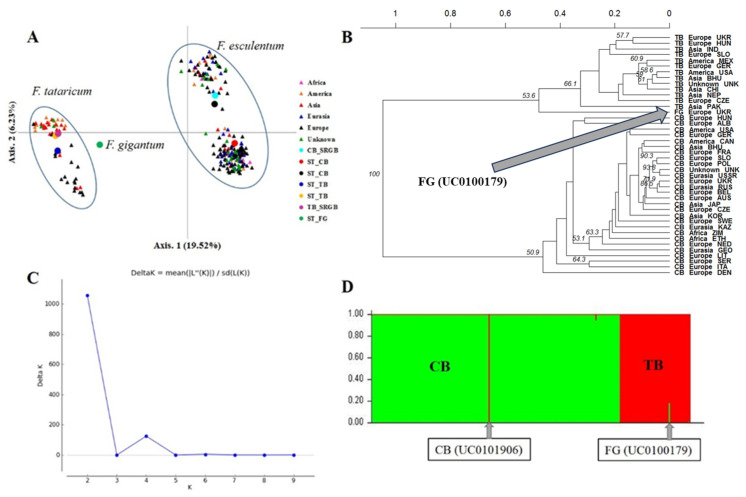
Patterns of the genetic differentiation between *F. esculentum* (CB) and *F. tataricum* (TB) accessions as represented by the first two principal coordinates of a PCoA (**A**) and UPGMA dendrogram based on Nei’s genetic distance (**B**). (**C**) The optimal number of clusters and (**D**) the estimated population structure of *F. esculentum* and *F. tataricum* accessions.

The pattern of genetic structure of buckwheat accessions was analysed using three different approaches. The 2D plot formed by the first two components of principal coordinate analysis (PCoA), which explained 19.52% and 6.23% of the genetic variation, respectively, showed that the *F. esculentum* and *F. tataricum* accessions were clearly divided into two genetically distinct groups across the first axis, while the second axis appeared to reflect genetic differentiation within the species (Figure 3A). The *F. esculentum* accessions could be subdivided into two major groups via the second axis, and the different regions in both groups were strongly intertwined. In *F. tataricum*, although the boundaries between regions were not clear, most of the European accessions appeared to be more variable and genetically distinct from the accessions from the other regions. In particular, the reference accession of *Fagopyrum* × *giganteum* UC0100179 (hybrid between common and Tartary buckwheat) appeared as an outgroup genetically close to *F. tataricum*. UPGMA clustering based on Nei’s genetic distance confirmed the genetic relationships among the analysed accessions and separated *F. esculentum* from *F. tataricum* with 100% bootstrap support (Figure 3B). However, the relatively low bootstrap values at the nodes separating geographic regions within each species (50.9% and 66.1%, respectively) indicate that the genetic structure of both species was not significantly affected by geographic origin (Figure 3B). The groups obtained by the PCoA plot and UPGMA clustering were identical to those inferred by Bayesian clustering performed with STRUCTURE. This showed that the ΔK plot peaked at K = 2 (Figure 3C), indicating the presence of two genetically distinct clusters that distinguish *F. esculentum* from *F. tataricum*. Strikingly, accessions of both species, including the six reference accessions, were assigned to one of the two clusters with membership coefficients (Q) greater than 0.94 for *F. esculentum* and 0.99 for *F. tataricum* (Figure 3D), with *F. esculentum* accession UC0101906 clustered with *F. tataricum* accessions with Q = 0.99, likely due to introgression with *F. tataricum*. Similar to the PCoA and UPGMA clustering results, the reference accession of *Fagopyrum* × *giganteum* UC0100179 was also clustered with the *F. tataricum* group with Q = 0.81 (Figure 3D).

**Figure 4:**
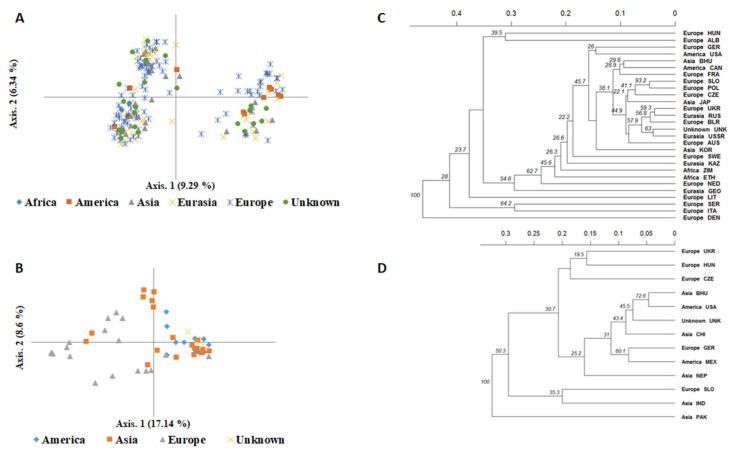
Relationships between *F. esculentum* (**A**) and *F. tataricum* (**B**) accessions as represented by the first two principal coordinates of a PCoA using allelic profiles from 24 SSR molecular markers. Samples are arranged based on their geographic origin. UPGMA dendrogram based on Nei’s genetic distance showing relationships between *F. esculentum* (**C**) and *F. tataricum* (**D**) accessions based on data from 24 SSR loci.

**Figure 5:**
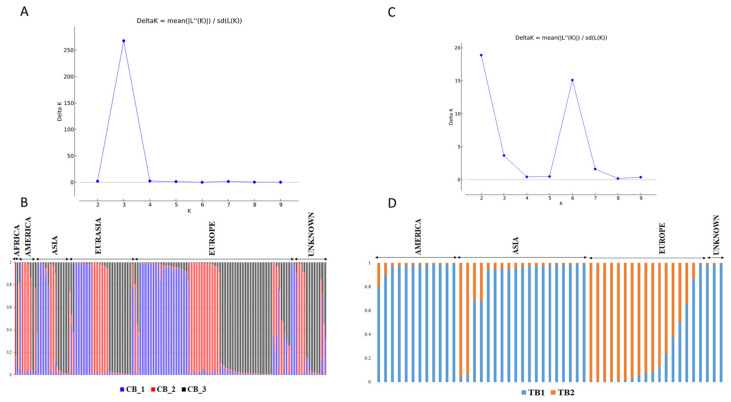
Results of the STRUCTURE Bayesian clustering based on the data of the 24 SSR markers. (**A**) The optimal number of clusters and (**B**) the estimated population structure of *F. esculentum* accessions. (**C**) The optimal number of clusters, and (**D**) the estimated population structure of *F. tataricum* accessions.

Further PCoA analysis, UPGMA clustering, and Bayesian clustering were performed to examine in detail the pattern of genetic structure within each species. For *F. esculentum*, PCoA analysis and UPGMA clustering were not geographically clustered (Figure 4A,C), suggesting that regional origin was not the main source of variation. The STRUCTURE clustering confirmed this pattern, dividing *F. esculentum* accessions into three main groups (optimal K = 3; Figure 5A) that were not associated with regional strata. Specifically, 164 accessions (86%) were assigned to a specific cluster with a membership coefficient (Q > 80%) (Figure 5B). The first cluster (CB1) consisted of 30 European accessions, eleven from Eurasia, seven from Asia, one from America and four from unknown regions. The second cluster (CB2) included 19 accessions from Europe, eight from Eurasia, four from Asia, six from America, and five from unknown regions, while the third cluster (CB3) included 32 European accessions, 14 from Eurasia, eight from Asia, two from America, three from Africa, and nine with unknown geographic origin. For *F. tataricum*, PCoA and UPGMA clustering also showed that the European accessions tended to be separated from the other accessions (Figure 4B,D). This pattern was also confirmed by Bayesian clustering, where *F. tataricum* accessions were divided into two main clusters (optimal K = 2; Figure 5C). The first cluster (TB1), which included 32 accessions (Q ≥ 80%), was mainly a mixture of American and Asian accessions (12 and 15 accessions, respectively), those of unknown geographic origin (three accessions), and only two accessions from Europe. The second cluster (TB2) included eleven European accessions and only two from Asia (Figure 5D).

### 2.5. Linkage Disequilibrium and Marker-Trait Association Analysis

**Figure 6:**
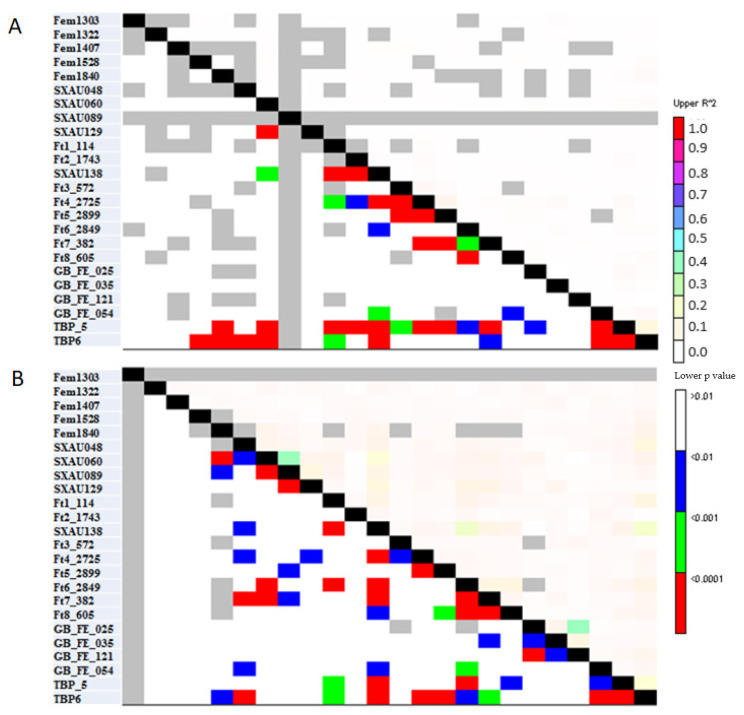
Pairwise linkage disequilibrium (LD) (*r*^2^) (lower diagonal) between the 24 SSR markers and *p*-value of *r*^2^ (upper diagonal) in (**A**) *F. esculentum* and (**B**) *F. tataricum*.

Linkage disequilibrium analysis (LD) in *F. esculentum* revealed 4770 pairwise comparisons among the 24 loci. The association between pairs of loci (*r*^2^) ranged from 0.001 to 0.07, with an average of 0.01, indicating a very low LD. One hundred and eighty loci pairs had *r*^2^ > 0.01, while only thirty pairs were in significant linkage disequilibrium at *p* < 0.001 (Figure 6A). In *F. tataricum*, *r*^2^ varied from 0.001 to 0.39 and averaged 0.04. Most loci pairs had *r*^2^ > 0.01, and only thirty-one loci pairs were significantly in LD at *p* < 0.001. Remarkably, a strong LD level was found between some SSR loci such as GB_FE_121 and GB_FE_025 (*r*^2^ > 0.39; *p* < 0.001) and SXAU089 and SXAU060 (*r*^2^ > 0.39; *p* < 0.001) (Figure 6B).

Association analysis between 24 SSRs and the 37 agro-morphological was performed using both a mixed linear model (MLM) and a general linear model (GLM). A total of 93 significant marker-trait associations (MTAs) were detected in *F. esculentum* using both approaches at the *p* < 0.05 probability level (Table S5). The 73 MTAs involving 22 SSR markers and 25 agro-morphological traits were identified with the GLM, 27 MTAs involving 14 SSR markers and 14 traits were identified with the MLM, while only six MTAs involving four SSRs and four traits were common to both models (Table S5). The phenotypic variation explained by each marker (*R*^2^) varied from 7.78% to 49.59% in GLM and from 7.49% to 44.60% in MLM, with an average across all markers of 24.99% and 24.67%, respectively, in both models. In GLM, the highest number of MTAs was recorded for seed length (eleven), followed by seed colour and plant growth habit (seven), while seven traits had a single MTA, such as germination duration, days to flowering, plant height, cyme length, and leaf blade shape. In the MLM, the highest number of MTAs (four) was associated with branched inflorescences and seed length, while the lowest number (one) of MTAs was recorded for eight traits such as number of cymes per plant, number of flower clusters per cyme, stem diameter, stem anthocyanin coloration, seed width, and 1000-seed weight. We also found that most SSR markers were simultaneously associated with multiple traits (multi-trait MTAs). The highest number of associations was recorded for Ft4_2725 (ten traits), followed by Ft1_114 and Ft3_572 (eight traits), Ft5_2899 and Ft8_605 (seven traits). The strongest associations were observed for the number of nodes in the stem (SNN) with Ft3_572 (*p* = 5.34 × 10^−8^; *R*^2^ = 42.82%), SXAU138 (*p* = 3.68 × 10^−4^; *R*^2^ = 42.87%) and TBP_6 (*p* = 1.81 × 10^−4^; *R*^2^ = 13.68%), and for the branched inflorescence (BINF) with Ft5_2899 (*p* = 1.40 × 10^−5^; *R*^2^ = 31.10%) (Table S5).

In *F. tataricum*, GLM and MLM identified 119 and 21 MTAs, respectively (*p* < 0.05), with 22 markers associated with 27 traits and 16 SSRs associated with 21 traits. Among all MTAs detected, there were only five overlapping associations derived from both models (Table S6). In the GLM model, the number of SSR loci significantly associated with each trait (MTA) was highest for the number of nodes in the stem (eleven), followed by 1000-seed weight and branched inflorescence (eight), and lowest for leaf blade length, number of leaves, anthocyanin colouration of the stem, and stem diameter (one). The proportion of phenotypic variation explained by each MTA (*R*^2^) ranged from 6.96% between SXAU048 and seed shape to 73.18% between Ft4_2725 and seed width, with an average of 31.74%. On the other hand, MLM analysis revealed that seed shape was associated with the highest number of loci (four), followed by number of flower clusters per cyme (three), seed colour (two), and number of nodes per stem (two). Among the detected MTAs, the *R*^2^ varied from 14.68% for the association between Fem1322 and germination duration to 74.08% for the association between Fem1840 and seed shape (Table S6). Notably, except for marker TBP_6, which was associated with the number of flower clusters per cyme (NFCC), all markers were found to be significantly associated with at least two phenotypes. The highest number of MTAs was observed for Ft5_2899 and Ft7_382, which were associated with 12 traits and explained 24.00–42.36% and 23.06–51.72% of the phenotypic variation in each of the associated traits, respectively. Fem1840 was associated with eleven traits and explained 14.17–74.08% of their phenotypic variation, and Ft4_2725 was associated with ten traits and explained 27.24–73.18% of their phenotypic variation. It should also be noted that among the strongest MTAs detected in *F. tataricum*, the number of nodes in the stem was firmly associated with Ft2_1743 (*p* = 9.35 × 10^−12^; *R*^2^ = 32.04%) and Ft6_2849 (*p* = 8.02 × 10^−6^; *R*^2^ = 27.71). Seed width showed a very strong relationship with GB_FE_054 (*p* = 2.24 × 10^−8^; *R*^2^ = 64.39%), TBP_5 (*p* = 4.66 × 10^−7^; *R*^2^ = 49.77%), and Ft4_2725 (*p* = 6.57 × 10^−5^; *R*^2^ = 73.18%). A very strong relationship was found between days of flowering and Ft6_2849 (*p* = 8.02 × 10^−6^; *R*^2^ = 27.71%). Seed colour was strongly associated with SXAU129 (*p* = 1.02 × 10^−5^; *R*^2^ = 57.09%) and Ft2_1743 (*p* = 1.38 × 10^−5^; *R*^2^ = 39.72%) (Table S6).

### 2.6. Selection of Representative Core Set Collections

Based on the analysis of SSR and agro-morphological data with PowerCore, a subset of 23 accessions was selected to form a core collection of *F. esculentum* (12.11% of the total collection), comprising 13 accessions from Europe, six from Eurasia, three from Asia, and one with unknown origin. At the molecular level, the retention rates of Ar, Na, Ho, and He between the core collection and the original collection were 63.97%, 66%, 97.70%, and 100%, respectively. At the agro-morphological level, the coincidence rate (CR %) and the variable rate of the coefficient of variation (VR %) for the 12 quantitative traits were 87.51% and 129.90%, respectively, while the class coverage of the 26 qualitative traits was 91.85%. For *F. tataricum*, the defined core collection consisted of 13 accessions (25.49% of the original collection), of which five were from Europe, four from Asia, three from America, and one accession of unknown origin. In this core set, 87.36%, 88.24%, 99.15%, and 100% of Ar, Na, Ho, and He, respectively, remained from the original collection. In addition, CR, VR and CC of the preserved collection are 85.63%, 108.56% and 97.11%, respectively.

## 3. Discussion

Optimal use and management of plant genetic resources and identification of accessions suitable for breeding programs rely on thorough analysis of genetic variation and structure of germplasm collections stored in genebanks [28]. The two buckwheat species *Fagopyrum esculentum* Moench (common buckwheat) and *F. tataricum* (L.) Gaertn. (Tartary buckwheat) are cultivated in various countries as dual-use pseudocereals with excellent nutritional and medicinal value [12]. Currently, over 10,000 different buckwheat accessions are deposited in various genebanks worldwide [13]. However, the genetic diversity of the available germplasm is poorly characterised, which hinders the use and selection of elite accessions for breeding programs [9]. Indeed, most previous studies have been conducted in local collections from hotspot regions such as China [29], India [30,31,32], Nepal [33], and Korea [34,35]. Some other studies were conducted in global collections but used only morphological traits [18,36] or only molecular tools such as AFLP [37], SSR markers [38] and genotyping by sequencing (GBS) [39]. To overcome these limitations, a large set of agro-morphological traits and microsatellite (SSR) markers were used to investigate the genetic diversity and structure of a comprehensive global collection of common and Tartary buckwheat and to perform marker-trait association analysis to identify candidate markers that modulate important agronomic traits that can be used for marker-assisted breeding of buckwheat.

Our results indicate that the buckwheat germplasm analysed in our study exhibits great agro-morphological diversity that can be used to select elite accessions for future breeding programmes. The diversity observed in our study was similar to that observed under field conditions for common and Tartary buckwheat collections from China [29], Nepal [17,40], India [30,41,42] and Japan [43]. A high level of diversity was also observed in some global collections [36,44,45]. Strikingly, in agreement with most of the above studies, relatively low variability was observed in days to flowering and seed-related traits such as seed length and width, seed surface, and seed shape. This pattern was expected since it has been reported that buckwheat accessions were mainly selected for early flowering [46,47] and large seed size [48] during the domestication process. However, similar to previous reports [23,49], considerable variation was observed in the seed colour, especially in common buckwheat. Such great variability could be associated with the environmental factors as the large collection of common buckwheat analysed in our study originated from diverse regions worldwide. In accordance, Oomah et al. [50] suggested that the seed colour in buckwheat is highly influenced by environmental variation. Importantly, Bulan et al. [51] have demonstrated that the seed colour was highly associated with maturity, with dark seeds characterised by early maturity. The same authors have reported that seed colour was commonly used to differentiate buckwheat accessions, indicating its importance in buckwheat domestication. Hence, the considerable variability revealed in common buckwheat collection analysed in our study suggests its relevance for future selection/breeding programs based on seed colour. Moreover, in agreement with the results of Joshi [40] and Kapoor et al. [42], similar trait association patterns were observed in both species. Our results were also in agreement with previous studies and showed that 1000-seed weight, which is an important trait for buckwheat breeding, was negatively associated with days to flowering and days to maturity [18,42]. On the other hand, similar to Tetsuka and Uchino [43], we found that 1000-seed weight had a high positive correlation with seed width and length, which is beneficial for selecting high-yielding elite accessions [52]. Moreover, our results showed significant phenotypic differentiation between the two species, and in agreement with previous studies [30,50,51], flower and seed traits were the main source of differentiation. This differentiation could be mainly due to the different pollination modes, as common buckwheat is self-incompatible with dimorphic flowers, while Tartary buckwheat is self-pollinated and homostylous [53]. In addition, it has been reported that the two species differ greatly in their growing areas because they are adapted to different environmental conditions. Compared to common buckwheat, Tartary buckwheat shows better adaptation to higher elevations and severe environmental conditions [48], which is due to its ability to develop various specific physiological, metabolic, and molecular responses reflected at the morphological level [2,53,54,55]. Moreover, at the intraspecific level, our results were consistent with previous studies based on agro-morphological traits [47,56] and seed nutrient profile [4] and showed that accessions were not geographically clustered. According to Chrungoo et al. [46], the lack of geographic structure in *Fagopyrum* species could be due to various factors such as massive seed exchange, spontaneous variation, genetic drift, natural selection, and artificial selection through the domestication process.

Previous studies have shown that genotype–environment interactions significantly affect the agro-morphological variation in buckwheat [47,57], indicating the great influence of the environment on the stability of traits that can affect the patterns of the genetic structure of the analysed germplasm. The use of DNA markers that are unaffected by the environment has been shown to be an efficient approach to characterise genetic diversity. Among these, simple sequence repeats (SSRs) are commonly used for such studies due to their high polymorphism, reproducibility, codominance, inheritance, transferability to other species, and great utility in marker-trait association [58,59]. Several SSR markers have been developed and used to characterise the genetic diversity of common [60,61,62,63] and Tartary buckwheat [38,64,65]. Importantly, Hou et al. [27] developed a large set of SSR markers through genome-wide screening and used them for genetic diversity analysis and marker-trait association to identify genomic regions associated with important agronomic traits in Tartary buckwheat. In our study, 25 SSR markers selected from the above studies were used to investigate the genetic diversity and structure of a large global collection of common and Tartary buckwheat. All markers analysed were transferable between common and Tartary buckwheat and were highly polymorphic, except for the marker Fem-1303, which was monomorphic in Tartary buckwheat. In a previous study, Bashir et al. [23] showed that of the 15 SSR markers developed for common buckwheat, only seven SSRs were transferable to Tartary buckwheat, all of which were included in our study. Remarkably, the average PIC value for the polymorphic SSRs used in our study was higher than the values reported for common [27,29,66] and Tartary buckwheat [23,27], indicating the high utility of SSR markers in analysing the genetic diversity of both species. However, the Fem1303 locus was found to be highly polymorphic in Tartary buckwheat [23,24], while previous studies revealed its prominent polymorphism in common buckwheat [25,66]. Importantly, Iwata et al. [63] showed that this locus is associated with flowering time in buckwheat. Considering the very low variability in days to flowering observed in our study for both species and the relatively low gene diversity maintained by Fem1303 in common buckwheat, we suspect that our analysed germplasm was subject to some selection for early-flowering accessions, resulting in the allelic fixation of this genomic region in Tartary buckwheat. Nevertheless, our results showed that the genetic diversity of common buckwheat (He = 0.77) was significantly higher than that of Tartary buckwheat (He = 0.66), and both values were higher than those previously reported for either species [27,35,66]. This pattern was expected because common buckwheat is an outcrossing species, whereas Tartary buckwheat is self-compatible [53]. In addition, the analysed collections for both species were originally from different regions of the world, whereas the collections used in the above studies were from restricted regions, which may explain the higher level of genetic diversity in our study. In agreement with our agro-morphological data and with previous studies using molecular markers such as RAPD [50,67] and SSR [23,47], our SSR data also showed clear genetic differentiation between common and Tartary buckwheat, confirming cross incompatibility between the two species. However, within species, the level of genetic diversity was not significantly different between regions and accessions were not geographically structured, indicating weak genetic differentiation between regions. The absence of such clustering suggests that strong natural selection has occurred continuously, resulting in continuous diversity. Consistent with previous studies using SSR markers in common [68,69] and Tartary buckwheat [27,29], it is hypothesised that the weak genetic differentiation is the result of extensive local and international seed exchange and/or recent extensive cultivation and selection.

The high genetic diversity and weak population structure observed in the common and Tartary buckwheat collections, as well as the low linkage disequilibrium among the SSR markers analysed, indicate that the germplasm studied and the results obtained meet the requirements for marker-trait association analysis useful for marker-assisted breeding. In common and Tartary buckwheat, few studies focused on identifying molecular markers associated with agronomic traits [1,27,60,70]. In this study, GLM and MLM models were used to identify many MTAs in both species. In both species, various agronomic traits were associated with many markers, indicating complex genetic control of these traits, especially seed yield and quality traits such as seed width, seed colour, seed length, seed shape, and 1000-seed weight, which are among the most important agronomic traits in buckwheat. Consistent with this, previous studies in many crops have shown that while seed traits are strongly influenced by the environment, their control is genetically complex [49,71,72,73]. Similar results were also obtained in Tartary buckwheat for 1000-seed weight and seed width [1,29,70,74]. Flowering time and plant height are also important traits that affect crop yield and plant architecture [61]. In our study, we found that days to flowering in common buckwheat were associated only with the marker Fem1840, which is one of the SSRs previously identified as flowering-associated markers [63], while this trait was strongly associated with Ft6_2849 in Tartary buckwheat (*p* = 7.03 × 10^−6^). These results suggest that genetic control of flowering time is very simple in both buckwheat species, making it an important trait for selection and breeding programs. Similarly, we found that plant height was associated with Ft1_114 only in common buckwheat. In Tartary buckwheat, the same marker was strongly associated with plant height and explained > 65% of the variation. This result suggests that this marker may be an optimal locus for marker-assisted breeding in both buckwheat species. In addition, our study identified numerous SSRs with pleiotropic association with seed traits and stem-related traits, providing a genetic explanation for the strong positive correlations between these traits. However, since MTAs are affected by environmental fluctuations [61], the strength of these associations should be tested by experimentation over multiple years/environments. To facilitate the feasibility of these trials and analyses and to accelerate breeding programs, a representative core collection should be established that covers the maximum agro-morphological and genetic diversity of the original germplasm. According to Joshi et al. [13], these core collections of ex situ accessions will be useful to decipher the genetic potential of buckwheat germplasm and develop nutrient-rich varieties around the world. In a recent study, an original Chinese collection of 1287 buckwheat accessions was characterised with phenotypic and SSR markers to develop a primary core collection of 530 accessions (41.18%) [60]. To our knowledge, this was the first attempt to define a core collection for buckwheat, and the developed collection was quite oversized and, therefore, difficult to handle. In our study, 23 and 13 accessions with multiple origins representative of buckwheat germplasm throughout the world (Europe, Asia, Eurasia, and America) were selected to create core collections for common and Tartary buckwheat, respectively. The two core collections included a reduced number of accessions, making them easily manageable, and captured most of the agro-morphological and genetic diversity of the original germplasm, indicating their great utility as a rich source of genetic diversity that can be used in buckwheat breeding programs. To that end, our data on the large worldwide collection provided a detailed characterisation of the genetic diversity based on agro-morphological traits and SSR markers of the two main cultivated buckwheat species. The findings of our study will contribute to the improvement of important agronomic traits and genomic regions. Controlling relevant traits identified will promote the efficient use of genetic and genomic resources for future molecular breeding of buckwheat. With the implementation of core collections for both species, we propose that efforts should be made to reduce the number of accessions for deeper molecular analyses in order to decode the genomic control of desired agronomic traits. In parallel, we encourage the continued collection, genetic characterisation, germplasm sharing, and long-term archiving of seed resources for future efficient use of buckwheat as a valuable food source.

## 4. Materials and Methods

### 4.1. Plant Material and Phenotyping Trial

The plant material used for the study consisted of the seeds of 190 accessions of *F. esculentum* and 51 accessions of *F. tataricum* (Table S1). The germplasm panel included 216 accessions originating from 36 different countries in Europe (Slovenia, Germany, Ukraine, Czech Republic, Poland, Hungary, Austria, Belarus, Italy, Lithuania, France, Sweden, Belarus, Slovakia, Serbia, Denmark, Albania, Netherlands), Eurasia (Russian Federation, Former Soviet Union, Kazakhstan and Georgia), Asia (Bhutan, China, Nepal, Japan, People’s republic of Korea, Pakistan and India), Africa (Ethiopia, Zimbabwe), and America (United States, Mexico, Canada), and 25 accessions with unknown origin. In addition, three accessions of *F. esculentum*, three accessions of *F. tataricum*, and one accession of *Fagopyrum x giganteum* were included for reference. For the phenotypic characterisation, five seeds (*n* = 5) per accession were grown to maturity during the 2021 growing season at the Agricultural Institute of Slovenia in Ljubljana, Slovenia (46°06′ N 14°51′ E, 295 m a.s.l.). Seeds were sown on May 10 in 7-L plastic pots filled with Potgrond H substrate (Klassman, Germany) in a glasshouse heated to 22 °C. One month later, the pots were moved outdoors. All accessions were phenotyped for 12 quantitative (Table 1) and 25 qualitative (Table S2) agro-morphological traits characterised as descriptors for buckwheat (*Fagopyrum* spp.) according to the International Plant Genetic Resources Institute (IPGR) [75] and the International Union for the Protection of New Varieties of Plants (UPOV) [76]. For phenotyping, the values of individual plants (*n* = 5) were used to calculate the mean values of the accessions for the quantitative traits, and a representative plant was selected to characterise the accession for the qualitative traits.

### 4.2. DNA Extraction and Molecular Analysis

Genomic DNA was extracted from a young, healthy leaf tissue using the DNeasy Plant Pro kit (Qiagen, Hilden, Germany) without the addition of PS buffer at the homogenisation step, as described in Pipan et al. [77]. Extracted DNAs were checked for quality and quantity using a fluorimeter (Qubit 3.0; ThermoFisher Scientific, Waltham, MA, USA) and diluted to a final concentration of 12.6 ng/μL. A set of 24 genus-specific SSR markers was used to investigate the genetic diversity of the studied accessions. The markers were selected based on their transferability, high polymorphism, and representative distribution among genomes and are, therefore, originating from different denomination groups: *Fem* [63] (five markers); *SXAU* [38] (five markers); *Ft* [67] (eight markers); *GB-FE* [65] (four markers); and *TBP* [68] (two markers) (Table S3). Depending on each primer pair, PCR reactions were performed under touch-down conditions as described in Pipan and Meglič [78]. Fragment analysis was performed on a genetic analyser (3500; Applied Biosystems, Waltham, MA, USA), and allele sizes were determined by comparison with an internal size standard (GeneScan-500 ROX; Applied Biosystems) using GeneMapper 6.0 software (Applied Biosystems).

### 4.3. Data Analysis

#### 4.3.1. Agro-Morphological Diversity

To evaluate the variability within and between the common and Tartary buckwheat species, the data of agro-morphological traits were analysed by univariate and multivariate approaches using the statistical programming environment R version 3.4.4 (https://www.r-project.org/, accessed on 13 March 2023). The package “*pastecs*” [79] was first used to calculate the maximum and minimum values (Max and Min), mean, standard deviation (SD) and coefficient of variation (CV) for the 12 quantitative traits. For the qualitative traits, the package “*diverse*” [80] was used to calculate the frequency of distribution and estimate their diversity level using the Shannon–Weaver diversity index [81]. Differences between the two species on quantitative and qualitative traits were tested using one-way analysis of variance (ANOVA) and Fisher’s Exact, respectively, implemented in the “*stats*” package with *p* ≤ 0.05 as the significance threshold. The association between pairwise analysed traits was estimated via Spearman correlation coefficient (ρ) using the package “*CorrPlot*” [82]. In addition, we used the packages “*FactoMiner*” and “*Factoextra*” [83] to perform a multifactorial analysis (MFA) to investigate the patterns of agro-morphological differentiation among all analysed accessions and to determine the main traits contributing to the differentiation between common and Tartary buckwheat accessions. The MFA results were plotted in a 2D plot formed by the two dimensions using the *fviz_mfa ()* function, and the contribution of each trait to variation among accessions was graphically presented based on each contribution to the first three MFA dimensions. The same packages were then used to perform further MFA analyses on the data for each species, and hierarchical clustering based on principal components (HCPC) was applied to the scores of the three MFA dimensions to divide the accessions into phenotypic groups (phenoclusters) with similar agro-morphological traits using the function *fviz_dend ()*.

#### 4.3.2. SSR Data Analysis

SSR data for both species were first analysed using PowerMarker version 3.25 [84] in order with the default settings to compute genetic diversity parameters, including the number of alleles (Na), major allele frequency (MAF), observed and expected heterozygosity (Ho and HE, respectively), the polymorphism information content (PIC) and fixation index (F). The allelic richness (Ar) was also computed using the R package “*Hierfstat*” [85]. Variation in Na, Ar, He, Ho and Fis was analysed with ANOVA implemented in the package “*stats*” to test the differences in genetic diversity between species and regions within species, with *p* ≤ 0.05 as the significance threshold. Moreover, we used the functions “*amova.test*” and “*amova.result*” to perform an analysis of molecular variance (AMOVA) using the R package “*poppr*” [86] on the SSR data of all common and Tartary buckwheat accessions to partition the genetic variation within and between species and to assess the influence of the region and the country of origin on the genetic variation within the species. Further AMOVA analyses were conducted on the datasets of each species separately in order to investigate the influence of the region and the country of origin on the genetic variation within the species. The significance of the explained variances was assessed using 999 permutations via the R package “*ade4*” [87]. Furthermore, in order to elucidate the genetic differentiation between the two species as well as the genetic relationship between the accessions within each species, we performed an unweighted pair group method arithmetic average (UPGMA) cluster analysis based on Nei’s genetic distance and principal coordinate analysis (PCoA) using the R package “*poppr*” and GenAlex v.6.5.2 [88], respectively. The accuracy of UPGMA clustering patterns was tested through 1000 bootstraps resamples using the ‘*poppr*’ package [86]. Additionally, STRUCTURE 2.3.4 [89] was used to conduct Bayesian clustering analyses without a priori classification of accessions in order to test whether *F. esculentum* and *F. tataricum* were genetically differentiated and to infer the genetic structure pattern at the species level. Each Bayesian clustering analysis was performed with the admixture model and the number of inferred clusters (K) set as 1–10, with the application of a burn-in period of 10,000 steps followed by 100,000 Monte Carlo Markov Chain replicates for each independent run. STRUCTURE outputs were then analysed using Structure Harvester v0.6.94 [90] based on Evanno’s approach [91] to determine the optimal number of clusters.

#### 4.3.3. Linkage Disequilibrium Evaluation and Marker-Traits Associations (MTAs) Analysis

For each species, the linkage disequilibrium (LD) in its significance between pairwise SSR markers was evaluated through the squared allele frequency correlations (*r*^2^) using the program TASSEL version 2.1 [92]. The same program was then applied to assess the association between agro-morphological traits and SSR markers (MTAs) in each species using (GLM) and the mixed linear model (MLM). The population structure (Q matrix) retrieved from STRUCTURE analysis was included as a covariate in the GLM model, while both the Q matrix and kinship (K) matrix, generated using TASSEL, were used in MLM. By applying the Bonferroni correction, the significance threshold for associations between traits and markers was set at *p* < 0.05, and the marker R-square (*R*^2^) was used to estimate the amount of variation explained by the SSR locus.

#### 4.3.4. Implementation of the Core Collections

Both the SSR and the agro-morphological data were used to develop representative core collections from the original collections of *F. esculentum* and *F. tataricum* in two steps. In the first step, the SSR data were first processed using PowerCore software 4.3.3 [93] by implementing an advanced M (maximisation) strategy to select the accessions that retain the maximum of the allelic diversity. In the second step, the agro-morphological data of the selected accessions were analysed using the same software to select subsets of accessions to implement final core sets for both species. The genetic diversity indices (Ar, Na, Ho, He, and Fis) were calculated to evaluate the retained genetic diversity in both core sets. In addition, the coincidence rate (CR %), the variable rate of coefficient of variation (VR%) and the class coverage (CC %) [93] were used to evaluate the agro-morphological diversity retained by the core sets.

## 5. Conclusions

This work has provided a deep insight into the agro-morphological and genetic diversity and structure of a worldwide collection of the two cultivated buckwheat species, *F. esculentum* Moench (common buckwheat) and *F. tataricum* (L.) Gaertn. (Tartary buckwheat) were given. The obtained results showed that the analysed collections have a great genetic diversity that can be used in future breeding programs. The genetic diversity of common buckwheat was significantly higher than that of Tartary buckwheat. However, for both species, this diversity was not significant between regions, indicating frequent seed exchange and/or recent extensive cultivation and selection. In addition, LD was very low between the SSR markers analysed, which is a desirable feature for using this germplasm panel for association studies and deciphering genetic control of agronomic traits of interest. The variation in agro-morphological traits analysed in our study was controlled by many genomic regions with pleiotropic and multipolygenic effects. To further explore the genetic potential of the buckwheat germplasm studied, core collections were established for both species for further genetic studies and future breeding efforts.

## Figures and Tables

**Table 1:** Summary of descriptive statistics for the 12 quantitative traits of *F. esculentum* and *F. tataricum*.

Trait	*F. esculentum*				*F. tataricum*				Differences
	Min	Max	Mean ± SE	CV (%)	Min	Max	Mean ± SE	CV (%)	
Germination period (days)	5.00	11.00	7.40 ± 0.09	16.88	7.00	11.00	7.54 ± 0.15	14.01	NS
Days to flowering	45.00	66.00	54.07 ± 0.28	7.26	51.00	66.00	58.38 ± 0.5	6.26	***
Plant height (cm)	57.60	143.00	105.18 ± 1.12	14.70	64.00	123.00	92.98 ± 2.16	16.73	***
Number of flower clusters per cyme	1.80	17.60	10.59 ± 0.2	26.07	1.60	20.80	13.98 ± 0.5	26.09	***
Number of cymes per plant	3.60	9.00	6.07 ± 0.07	16.89	5.80	12.80	8.56 ± 0.19	15.71	***
Length of cyme (cm)	18.80	92.00	59.5 ± 0.83	19.40	17.00	93.00	64.08 ± 1.79	20.25	**
Petiole length (mm)	4.33	11.57	7.05 ± 0.09	17.90	5.45	11.25	8.11 ± 0.16	14.39	***
Leaf blade length (cm)	5.22	11.20	7.97 ± 0.08	13.92	5.58	9.86	7.46 ± 0.14	13.58	***
Leaf blade width (cm)	5.52	12.64	8.39 ± 0.08	13.84	5.22	10.64	8.50 ± 0.20	17.23	NS
Seed length (mm)	4.43	7.23	6.11 ± 0.03	6.63	3.20	6.16	4.68 ± 0.07	11.59	***
Seed width (mm)	2.85	5.44	4.18 ± 0.03	10.45	1.98	4.55	2.99 ± 0.05	11.07	***
1000-seed weight (g)	13.56	38.70	25.42 ± 0.33	18.15	9.00	26.27	16.2 ± 0.45	20.02	***

Min—minimum; Max—maximum; SE—standard error; CV—coefficient of variation. ***, *p* < 0.0001; **, *p* < 0.001; NS—non significant.

**Table 2:** Genetic diversity parameters of the 24 SSR markers used for the molecular characterisation of *F. esculentum* and *F. tataricum* accessions.

Marker	*F. esculentum*							*F. tataricum*						
	MAF	Ar	Na	Ho	He	Fis	PIC	MAF	Ar	Na	Ho	He	PIC	Fis
Fem1303	0.65	8.21	12.00	0.55	0.54	−0.03	0.50	1.00	1.00	1.00	0.00	0.00	0.00	NA
Fem1322	0.18	10.67	16.00	0.98	0.89	−0.11	0.88	0.61	3.00	3.00	0.59	0.53	0.46	−0.10
Fem1407	0.37	11.60	20.00	0.91	0.80	−0.13	0.78	0.43	6.84	7.00	0.71	0.70	0.65	−0.01
Fem1528	0.19	13.93	18.00	0.92	0.88	−0.05	0.87	0.31	6.99	7.00	0.98	0.79	0.76	−0.24
Fem1840	0.29	11.45	15.00	0.80	0.84	0.05	0.82	0.24	9.00	9.00	0.60	0.83	0.81	0.29
SXAU048	0.21	15.47	25.00	0.83	0.88	0.05	0.87	0.41	5.00	5.00	0.84	0.73	0.70	−0.15
SXAU060	0.51	7.34	14.00	0.63	0.69	0.09	0.66	0.63	2.00	2.00	0.00	0.47	0.36	1.00
SXAU089	0.21	12.00	17.00	0.53	0.90	0.27	0.89	0.34	5.00	5.00	1.00	0.74	0.69	−0.36
SXAU129	0.23	10.50	17.00	0.94	0.89	−0.06	0.88	0.58	4.92	5.00	0.75	0.57	0.50	−0.31
SXAU138	0.26	7.48	14.00	0.92	0.84	−0.10	0.82	0.54	4.88	5.00	0.78	0.53	0.43	−0.47
Ft1_114	0.26	7.52	11.00	0.90	0.83	−0.09	0.81	0.44	8.76	9.00	0.96	0.70	0.65	−0.38
Ft2_1743	0.33	6.91	9.00	0.96	0.78	−0.23	0.75	0.50	2.99	3.00	0.96	0.52	0.40	−0.85
Ft3_572	0.24	9.72	13.00	0.98	0.87	−0.12	0.86	0.22	13.92	14.00	0.90	0.89	0.88	−0.02
Ft4_2725	0.24	7.55	13.00	0.86	0.81	−0.07	0.78	0.25	10.00	10.00	1.00	0.86	0.85	−0.16
Ft5_2899	0.32	6.77	13.00	0.95	0.81	−0.18	0.79	0.20	13.99	14.00	0.96	0.90	0.89	−0.07
Ft6_2849	0.42	5.64	13.00	0.82	0.73	−0.14	0.69	0.33	7.99	8.00	0.96	0.75	0.71	−0.29
Ft7_382	0.39	9.46	16.00	0.92	0.80	−0.15	0.78	0.49	5.92	6.00	0.45	0.67	0.62	0.33
Ft8_605	0.56	10.95	16.00	0.70	0.65	−0.07	0.64	0.31	7.99	8.00	1.00	0.79	0.76	−0.27
GB_FE_025	0.47	6.35	13.00	0.98	0.69	−0.42	0.65	0.46	5.00	5.00	0.94	0.64	0.57	−0.48
GB_FE_035	0.48	4.52	9.00	1.00	0.57	−0.75	0.48	0.44	6.99	7.00	1.00	0.69	0.64	−0.45
GB_FE_121	0.41	7.69	12.00	0.99	0.68	−0.45	0.63	0.54	3.00	3.00	0.92	0.56	0.48	−0.64
GB_FE_054	0.23	5.96	14.00	0.94	0.84	−0.12	0.82	0.32	7.00	7.00	0.90	0.80	0.77	−0.13
TBP_5	0.35	6.90	10.00	0.56	0.78	0.29	0.75	0.47	5.00	5.00	0.53	0.68	0.63	0.22
TBP_6	0.63	2.78	6.00	0.06	0.51	0.89	0.43	0.64	4.92	5.00	0.06	0.53	0.47	0.89
Mean	0.35	8.64	13,95	0.82	0.77	−0.07	0.74	0,44	6.34	6.38	0.74	0.66	0.61	−0.12

MAF—major allele frequency; Ar—allelic richness; Na—number of alleles observed; Ho—observed heterozygosity; He—expected heterozygosity; PIC—polymorphism information content; Fis—fixation index. The bold marker Fem1303 was monomorphic for *F. tataricum* and was not included in the mean calculation.

**Table 3:** Genetic diversity parameters at the regions’ scale and the selected core collections *F. esculentum* and *F. tataricum* and summary of ANOVA testing the effects of species and region of origin on genetic diversity statistics.

		N	Ar	Na	Ho	He	Fis
*F. esculentum*	Africa	3	3.66	3.67	0.75	0.64	−0.15
	America	11	3.78	6.92	0.81	0.74	−0.10
	Asia	22	3.73	8.75	0.80	0.74	−0.07
	Eurasia	40	3.71	9.46	0.84	0.76	−0.11
	Europe	92	3.80	12.71	0.82	0.77	−0.07
	Unknown	22	3.81	8.75	0.83	0.76	−0.09
	Mean		3.75	8.38	0.81	0.74	−0.10
Core collection	23		8.63	9.21	0.83	0.78	−0.06
*F. tataricum*	Africa	0	0.00	0.00	0.00	0.00	0.00
	America	12	2.81	4.17	0.72	0.57	−0.26
	Asia	19	2.99	4.92	0.76	0.63	−0.17
	Eurasia	0	0.00	0.00	0.00	0.00	0.00
	Europe	18	3.09	5.75	0.73	0.66	−0.11
	Unknown	3	2.75	2.75	0.79	0.52	−0.55
	Mean		1.94	3.52	0.60	0.48	−0.22
Core collection	13		5.53	5.63	0.73	0.67	−0.09
**Species**	**F**	**3.706**	**9.159**	**28.028**	**3.986**	**8.425**	**0.855**
	***p***	**0.112**	***0.029***	***0.003***	**0.102**	***0.034***	**0.398**
**Region**	**F**	**1.584**	**1.135**	**3.738**	**1.119**	**1.443**	**0.786**
	***p***	**0.313**	**0.446**	**0.087**	**0.453**	**0.349**	**0.601**

N—sample size; Ar—allelic richness; Na—number of alleles observed; Ho—observed heterozygosity; He—expected heterozygosity; Fis—fixation index. ANOVA results are in bold values. Italic values are significant at *p* > 0.05.

**Table 4:** Analysis of molecular variance (AMOVA) and Monte-Carlo significance tests for the collection of 190 accessions of *F. esculentum* and 51 accessions of *F. tataricum*.

	DF	Sum of Squares	Mean Square	Variance Components	Total Variance (%)	*p*
Buckwheat collection						
Variations between species	1	920.35	920.35	5.36	23.83	0.001
Between accessions within species	240	2019.98	8.42	0.00	0.00	1.000
Within accessions	242	2326.50	9.61	9.61	76.17	0.001
*F. esculentum*						
Variations between regions	5	93.58	18.71	0.00	0.00	0.930
Variations between countries within the region	22	437.47	19.88	0.38	2.21	1
Between accessions within the country	162	2523.03	15.57	0.00	0.00	1
Within accessions	190	3631.01	19.11	19.11	97.79	0.0001
*F. tataricum*						
Variations between regions	3	99.92	33.30	0.38	2.45	0.220
Variations between countries within the region	9	149.16	16.57	1.01	6.53	1
Between accessions within the country	39	429.35	11.00	0.00	0.00	1
Within accessions	52	895.30	17.21	17.21	91.02	0.0001

DF—degree of freedom.

## Data Availability

The dataset supporting the reported results is available in the ECOBREED Zenodo community: https://doi.org/10.5281/zenodo.8102404 (accessed on 30 June 2023). This access is currently restricted but will be “open access” once the manuscript is accepted for publication.

## Supplementary Materials

The following supporting information can be downloaded at https://www.mdpi.com/article/10.3390/plants12183321/s1. Table S1: The accessions of common buckwheat (*F. esculentum*) and Tartary buckwheat (*F. tataricum*) included in this agro-morphological and molecular characterisation. Table S2: Descriptive statistics of the 37 qualitative traits in common buckwheat (*F. esculentum*) and Tartary buckwheat (*F. tataricum*). Table S3: List of the 24 SSR markers used for genetic diversity analysis of common buckwheat (*F. esculentum*) and Tartary buckwheat (*F. tataricum*). Table S4: Correlation matrices of the 37 agro-morphological traits based on Spearman’s coefficient in *F. esculentum* (above the diagonal) and *F. tataricum* (below the diagonal). Only significant correlations are shown (*p* < 0.01). Table S5: Marker-trait associations for common buckwheat (*F. esculentum*) generated by the general linear model (GLM) and mixed linear model (MLM) at *p* < 0.05. Table S6: Marker-trait associations for Tartary buckwheat (*F. tataricum*) generated by general linear model (GLM) and mixed linear model (MLM) at *p* < 0.05.
