# Supplementary material for: Agro-Morphological and Molecular Characterization Reveal Deep Insights in Promising Genetic Diversity and Marker-Trait Associations in Fagopyrum esculentum and Fagopyrum tataricum
Source: Plants (Basel). 2023 Sep 20;12(18):3321. doi: 10.3390/plants12183321 (PMC10534386; doi:10.3390/plants12183321)
Supplement: Supplementary file 1 [file plants-12-03321-s001.zip › plants-2608655-supplementary.pdf]

plants

MDPI

Supplementary Materials

Agro-Morphological and Molecular Characterization Reveal Deep Insights in Promising Genetic Diversity and Marker-Trait Associations in *Fagopyrum esculentum* and *Fagopyrum tataricum*

Table S1. The accessions of common buckwheat (*F. esculentum*) and Tartary buckwheat (*F. tataricum*) included in this agro-morphological and molecular characterization.

ID	Accession number	Accession name	Country of origin	Region	Species
32	01Z5000137	Pulawska II	Poland	Europe	*Fagopyrum esculentum*
11		KIS Doris	Slovenia	Europe	*Fagopyrum tataricum*
52	SRGB 2258	Dolina Krme na Gorenjskem	Slovenia	Europe	*Fagopyrum tataricum*
55	SRGB 2224	Osrednje Goričko	Slovenia	Europe	*Fagopyrum tataricum*
56	SRGB 2409	Straža	Slovenia	Europe	*Fagopyrum tataricum*
58	SRGB 2257	Radohova vas	Slovenia	Europe	*Fagopyrum tataricum*
59	SRGB 2321	Novo Mesto	Slovenia	Europe	*Fagopyrum tataricum*
66	SRGB 2337		Slovenia	Europe	*Fagopyrum tataricum*
68	SRGB 2304		Slovenia	Europe	*Fagopyrum tataricum*
72	SRGB 2356		Slovenia	Europe	*Fagopyrum tataricum*
77	PI 658438	Martin's Tarbary	United States	America	*Fagopyrum tataricum*
88	PI 658430 00GI SD	G 32049	United States	America	*Fagopyrum tataricum*
89	PI 476852	Madawaska	United States	America	*Fagopyrum tataricum*
90	PI 658429	G 32048	United States	America	*Fagopyrum tataricum*
122	01Z5100008	PI 481670	Bhutan	Asia	*Fagopyrum tataricum*
124	01Z5100014	PI 476852	United States	America	*Fagopyrum tataricum*
127	01Z5100003	PI 481646	Bhutan	Asia	*Fagopyrum tataricum*
128	01Z5100004	PI 481652	Bhutan	Asia	*Fagopyrum tataricum*
131	01Z5100010	Lifago	Germany	Europe	*Fagopyrum tataricum*
135	01Z5100037	PI 481661	Bhutan	Asia	*Fagopyrum tataricum*

143	01Z5100041	Jianzui	China	Asia	*Fagopyrum tataricum*
145	01Z5100044	Zhaoqiao-1	China	Asia	*Fagopyrum tataricum*
185	UC0101691	Peremoga	Ukraine	Europe	*Fagopyrum tataricum*
121	01Z5100007	PI 481659	Bhutan	Asia	*Fagopyrum tataricum*
123	01Z5100013	PI 451723	Mexico	America	*Fagopyrum tataricum*
126	01Z5100002	PI 481645	Bhutan	Asia	*Fagopyrum tataricum*
129	01Z5100006	PI 481658	Bhutan	Asia	*Fagopyrum tataricum*
130	01Z5100009	PI 481671	Bhutan	Asia	*Fagopyrum tataricum*
133	01Z5100019	RCAT 068749	unknown	Unknown	*Fagopyrum tataricum*
136	01Z5100050	Sarasin a Ployes	United States	America	*Fagopyrum tataricum*
141	01Z5100012		Czech Republic	Europe	*Fagopyrum tataricum*
146	01Z5100046	Jinqiao-2	China	Asia	*Fagopyrum tataricum*
147	01Z5100030	PI 427239	Nepal	Asia	*Fagopyrum tataricum*
184	UC0101690	Lira	Ukraine	Europe	*Fagopyrum tataricum*
108	01Z5000056	35273	Germany	Europe	*Fagopyrum esculentum*
3		Panda	Russian Federation	Eurasia	*Fagopyrum esculentum*
18	01Z5000143	CD 7272	Czech Republic	Europe	*Fagopyrum esculentum*
28	01Z5000112	GEMA	Poland	Europe	*Fagopyrum esculentum*
95	PI 658441 02GI SD	PA 001	United States	America	*Fagopyrum esculentum*
165	01Z5000124	Bongpyng	unknown	Unknown	*Fagopyrum esculentum*
196	UC0100149		Hungary	Europe	*Fagopyrum esculentum*
2		Zoe	Czech Republic	Europe	*Fagopyrum esculentum*
5		Kora	unknown	Unknown	*Fagopyrum esculentum*
6		HRUSZOWSKA	Poland	Europe	*Fagopyrum esculentum*
8		Billy	Austria	Europe	*Fagopyrum esculentum*
12		Zamira	unknown	Unknown	*Fagopyrum esculentum*
14	01Z5000071	Dozhdik	Belarus	Europe	*Fagopyrum esculentum*
19	01Z5000058	BALLADA	Former Soviet Union	Eurasia	*Fagopyrum esculentum*

24	01Z5000072	Sudtirol Nr. 3	unknown	Unknown	*Fagopyrum esculentum*
26	01Z5000017	MONORI	unknown	Unknown	*Fagopyrum esculentum*
30	01Z5000049	Yaita Zairai	Japan	Asia	*Fagopyrum esculentum*
31	01Z5000050	Stoyoama Zairai	Japan	Asia	*Fagopyrum esculentum*
37	01Z5000067	Chernigovskaya 17	Ukraine	Europe	*Fagopyrum esculentum*
38	01Z5000004	Chishiminskaya	Former Soviet Union	Eurasia	*Fagopyrum esculentum*
39	01Z5000006	Krasnostreletskaya	Former Soviet Union	Eurasia	*Fagopyrum esculentum*
41	01Z5000051	Botansoba	Japan	Asia	*Fagopyrum esculentum*
45	01Z5000063	PYRA	Czech Republic	Europe	*Fagopyrum esculentum*
46	01Z5000113	Lopfe	unknown	Unknown	*Fagopyrum esculentum*
48	01Z5000001	AELITA	Former Soviet Union	Eurasia	*Fagopyrum esculentum*
50	01Z5000013	Alaya 846	Ukraine	Europe	*Fagopyrum esculentum*
67	SRGB 2265	Gorenje Nekovo, Kanal	Slovenia	Europe	*Fagopyrum esculentum*
71	SRGB 2198	Vrhtrebnje	Slovenia	Europe	*Fagopyrum esculentum*
85	PI 658436	Martin's Alaska	United States	America	*Fagopyrum esculentum*
86	PI 647601	Nostrano	Italy	Europe	*Fagopyrum esculentum*
103	01Z5000118	Podojlanka,Podolyanka	Former Soviet Union	Eurasia	*Fagopyrum esculentum*
109	01Z5000057	53342	Germany	Europe	*Fagopyrum esculentum*
110	01Z5000062	Bolshevik 4	Former Soviet Union	Eurasia	*Fagopyrum esculentum*
111	01Z5000025	8429	unknown	Unknown	*Fagopyrum esculentum*
116	01Z5000012	Shatilovskaya 5	Former Soviet Union	Eurasia	*Fagopyrum esculentum*
150	01Z5000139	Silverhull 24	Canada	America	*Fagopyrum esculentum*
167	01Z5000084	Anita Beloruskaya	Ukraine	Europe	*Fagopyrum esculentum*
169	01Z5000014	UKRAJINSKAJA,Viktoriya Podolskaya	Former Soviet Union	Eurasia	*Fagopyrum esculentum*
170	01Z5000008	Maiskaya	Former Soviet Union	Eurasia	*Fagopyrum esculentum*
180	UC0101319		Ukraine	Europe	*Fagopyrum esculentum*
197	UC0100170	Krupnoplodnaya	Russian Federation	Eurasia	*Fagopyrum esculentum*
200	UC0100212	Mestnyy 31	Lithuania	Europe	*Fagopyrum esculentum*

205	UC0100281		France	Europe	*Fagopyrum esculentum*
207	UC0100283		Sweden	Europe	*Fagopyrum esculentum*
215	UC0100365	CHornogorets'	Belarus	Europe	*Fagopyrum esculentum*
217	UC0100948	MostĂŞapaz	Poland	Europe	*Fagopyrum esculentum*
226	UC0101992	Perlyna Podil's'ka	Ukraine	Europe	*Fagopyrum esculentum*
239	UC0102205	Feniks	Belarus	Europe	*Fagopyrum esculentum*
36	01Z5000066	Prego	Germany	Europe	*Fagopyrum esculentum*
158	01Z5000024	8428	unknown	Unknow n	*Fagopyrum esculentum*
216	UC0100946		United States	America	*Fagopyrum esculentum*
1		Zita	Czech Republic	Europe	*Fagopyrum esculentum*
10		KIS Čebelica	Slovenia	Europe	*Fagopyrum esculentum*
15	01Z5000010	Sibiryachka	Former Soviet Union	Eurasia	*Fagopyrum esculentum*
20	01Z5000141	Sweden-1	Sweden	Europe	*Fagopyrum esculentum*
34	01Z5000133	ROKSOLANA	Russian Federation	Eurasia	*Fagopyrum esculentum*
43	01Z5000060	Sumcanka	Former Soviet Union	Eurasia	*Fagopyrum esculentum*
73	SRGB 5702	SVNGOR 2010-14	Slovenia	Europe	*Fagopyrum esculentum*
75	SRGBTBA	HUNORS 2010-9	Slovenia	Europe	*Fagopyrum esculentum*
101	01Z5000064	La Harpe	France	Europe	*Fagopyrum esculentum*
113	01Z5000093	JEZYK	unknown	Unknow n	*Fagopyrum esculentum*
119	01Z5000032	PI 481632	Bhutan	Asia	*Fagopyrum esculentum*
151	01Z5000022	4411	unknown	Unknow n	*Fagopyrum esculentum*
161	01Z5000121	Rana 60	Slovenia	Europe	*Fagopyrum esculentum*
168	01Z5000015	Vita Galeya	Former Soviet Union	Eurasia	*Fagopyrum esculentum*
182	UC0101642	DETERMINANT 2	Ukraine	Europe	*Fagopyrum esculentum*
189	UC0101942		Austria	Europe	*Fagopyrum esculentum*
203	UC0100278		People's republic of Korea	Asia	*Fagopyrum esculentum*
213	UC0100362	Bilorus'ka gomostil'na	Belarus	Europe	*Fagopyrum esculentum*
227	UC0102165	Kvyetka determinantna	Belarus	Europe	*Fagopyrum esculentum*

241	63 working collection	Aiva	People's republic of Korea	Asia	*Fagopyrum esculentum*
243	66	From Poland	unknown	Unknown	*Fagopyrum esculentum*
33	01Z5000131	Ceska krajova	Czech Republic	Europe	*Fagopyrum esculentum*
53	SRGB 2316		Slovenia	Europe	*Fagopyrum tataricum*
54	SRGB 2223	Slovenj Gradec	Slovenia	Europe	*Fagopyrum tataricum*
83	PI 503879 90GI SD	Solider Pond	United States	America	*Fagopyrum tataricum*
87	PI 658431	G 32050	United States	America	*Fagopyrum tataricum*
96	PI 451723	Trigo Saraceno O'Alforfon	Mexico	America	*Fagopyrum tataricum*
132	01Z5100017	Weswod Ican	unknown	Unknown	*Fagopyrum tataricum*
138	01Z5100025	290	Bhutan	Asia	*Fagopyrum tataricum*
139	01Z5100001	PI 481644	Bhutan	Asia	*Fagopyrum tataricum*
142	01Z5100028	PA 160	unknown	Unknown	*Fagopyrum tataricum*
144	01Z5100042	Liuqiao-3	China	Asia	*Fagopyrum tataricum*
186	UC0101692	Kalyna	Ukraine	Europe	*Fagopyrum tataricum*
198	UC0100180		China	Asia	*Fagopyrum tataricum*
210	UC0100291		India	Asia	*Fagopyrum tataricum*
220	UC0101037		Hungary	Europe	*Fagopyrum tataricum*
234	UC0100179		Ukraine	Europe	*Fagopyrum giganteum*
44	01Z5000061	SKOROSPELAJA,SKOROSPELAYA	Former Soviet Union	Eurasia	*Fagopyrum esculentum*
64	SRGB 2222		Slovenia	Europe	*Fagopyrum esculentum*
162	01Z5000048	Takizawa Zairai	Japan	Asia	*Fagopyrum esculentum*
166	01Z5000125	Suwon #1	unknown	Unknown	*Fagopyrum esculentum*
187	UC0101906	mistsevii 58	Lithuania	Europe	*Fagopyrum esculentum*
212	UC0100723		Belarus	Europe	*Fagopyrum esculentum*
214	UC0100912	Serebrista	Ukraine	Europe	*Fagopyrum esculentum*
232	UC0102193	Vlada	Belarus	Europe	*Fagopyrum esculentum*
9		KIS Eva	Slovenia	Europe	*Fagopyrum esculentum*
22	01Z5000129	LUBA	Russian Federation	Eurasia	*Fagopyrum esculentum*

25	01Z5000140	Tempest	Canada	America	*Fagopyrum esculentum*
29	01Z5000046	Iwate Zairai (MIDOU)	Japan	Asia	*Fagopyrum esculentum*
40	01Z5000018	Lehnicka krajova	Slovakia	Europe	*Fagopyrum esculentum*
47	01Z5000059	KRUPINKA	Former Soviet Union	Eurasia	*Fagopyrum esculentum*
57	SRGB 2285	Kleče	Slovenia	Europe	*Fagopyrum esculentum*
60	SRGB 2296	Brusnice, Novo Mesto	Slovenia	Europe	*Fagopyrum esculentum*
61	SRGB 2207	Slovenj Gradec	Slovenia	Europe	*Fagopyrum esculentum*
63	SRGB 2305	Jeprca, Medvode	Slovenia	Europe	*Fagopyrum esculentum*
65	SRGB 2273	Vojščica pri Kostanjevici	Slovenia	Europe	*Fagopyrum esculentum*
76	PI 658444	Laharpe/PA 029	France	Europe	*Fagopyrum esculentum*
80	PI 647598	Pulawska/PA 054	Poland	Europe	*Fagopyrum esculentum*
93	PI 658446 99GI SD	Clfa 33	United States	America	*Fagopyrum esculentum*
97	PI 647673	Gornosorskaya	Former Soviet Union	Eurasia	*Fagopyrum esculentum*
98	PI 658447	PA 002	United States	America	*Fagopyrum esculentum*
102	01Z5000115	Kasanskaya	Former Soviet Union	Eurasia	*Fagopyrum esculentum*
106	01Z5000040	PI 481641	Bhutan	Asia	*Fagopyrum esculentum*
107	01Z5000074	832003	unknown	Unknown	*Fagopyrum esculentum*
115	01Z5000009	Prikamskaya	Former Soviet Union	Eurasia	*Fagopyrum esculentum*
117	01Z5000016	LOCAL	unknown	Unknown	*Fagopyrum esculentum*
118	01Z5000030	PI 481630	Bhutan	Asia	*Fagopyrum esculentum*
120	01Z5000117	Demetra	Former Soviet Union	Eurasia	*Fagopyrum esculentum*
149	01Z5000138	Tohno Zairai	Canada	America	*Fagopyrum esculentum*
152	01Z5000029	PI 481629	Bhutan	Asia	*Fagopyrum esculentum*
157	01Z5000023	4419	unknown	Unknown	*Fagopyrum esculentum*
159	01Z5000094	JUBILEJNAJA,Yubileina	unknown	Unknown	*Fagopyrum esculentum*
163	01Z5000076	Zelenocvetkovaya 90	unknown	Unknown	*Fagopyrum esculentum*
164	01Z5000005	Kalininskaya	Former Soviet Union	Eurasia	*Fagopyrum esculentum*
177	01Z5000042	PI 481643	Bhutan	Asia	*Fagopyrum esculentum*

178	UC0101155	KOSMEYA	Ukraine	Europe	*Fagopyrum esculentum*
179	UC0101182		Austria	Europe	*Fagopyrum esculentum*
190	UC0101948		Ethiopia	Africa	*Fagopyrum esculentum*
191	UC0101972	AKADEMICHNA	Ukraine	Europe	*Fagopyrum esculentum*
195	UC0100100	Hibridnaya	Russian Federation	Eurasia	*Fagopyrum esculentum*
199	UC0100210	Bogatyr	Russian Federation	Eurasia	*Fagopyrum esculentum*
204	UC0100279		People's republic of Korea	Asia	*Fagopyrum esculentum*
208	UC0100285	Tetraployidna	Russian Federation	Eurasia	*Fagopyrum esculentum*
209	UC0100280		France	Europe	*Fagopyrum esculentum*
211	UC0100361	Sadkom	Poland	Europe	*Fagopyrum esculentum*
221	UC0101129	Kara-Dag	Ukraine	Europe	*Fagopyrum esculentum*
223	UC0100988	Mig	Russian Federation	Eurasia	*Fagopyrum esculentum*
224	UC0101092	SKOROSTIGLA 86	Russian Federation	Eurasia	*Fagopyrum esculentum*
236	UC0101698	Krupnoplodna SHertandins'k	Kazachstan	Eurasia	*Fagopyrum esculentum*
247	01Z5000135	Bamby	Austria	Europe	*Fagopyrum esculentum*
248	01Z5000130	Billy	Austria	Europe	*Fagopyrum esculentum*
21	01Z5000070	Spacinska 1	Slovakia	Europe	*Fagopyrum esculentum*
62	SRGB 2266	Krajna vas, Sežana	Slovenia	Europe	*Fagopyrum esculentum*
78	PI 647640	CD 7272	Czech Republic	Europe	*Fagopyrum esculentum*
114	01Z5000105	Komsta	Former Soviet Union	Eurasia	*Fagopyrum esculentum*
231	UC0100177	Emka	Poland	Europe	*Fagopyrum esculentum*
233	UC0102207	Ruslana	Ukraine	Europe	*Fagopyrum esculentum*
4		Harpe	France	Europe	*Fagopyrum esculentum*
17	01Z5000052	Hara Zairai	Japan	Asia	*Fagopyrum esculentum*
27	01Z5000047	Tokushima Zairai	Japan	Asia	*Fagopyrum esculentum*
42	01Z5000069	Vychodoslovenska krajova	Slovakia	Europe	*Fagopyrum esculentum*
49	01Z5000007	Lada	Former Soviet Union	Eurasia	*Fagopyrum esculentum*
51	01Z5000111	Emka	Poland	Europe	*Fagopyrum esculentum*

69	SRGB 2270	Temnica na Krasu	Slovenia	Europe	*Fagopyrum esculentum*
74	SRGB 5553	SVNDOL2007-41	Slovenia	Europe	*Fagopyrum esculentum*
81	PI 263949	PA 127	Serbia	Europe	*Fagopyrum esculentum*
82	PI 647594	Tokyo/PA 011	Canada	America	*Fagopyrum esculentum*
91	PI 647638 12GI SD	MC 060	Former Soviet Union	Eurasia	*Fagopyrum esculentum*
92	PI 647603	Sarrasin du Pays/PA 030	France	Europe	*Fagopyrum esculentum*
94	PI 658440	Enka	Denmark	Europe	*Fagopyrum esculentum*
99	01Z5000011	Skorospelaya 81	Former Soviet Union	Eurasia	*Fagopyrum esculentum*
104	01Z5000002	Astoriya	Former Soviet Union	Eurasia	*Fagopyrum esculentum*
105	01Z5000037	PI 481638	Bhutan	Asia	*Fagopyrum esculentum*
112	01Z5000092	Kasanskaya,Kusanskaja	Belarus	Europe	*Fagopyrum esculentum*
125	01Z5000120	Darja	Slovenia	Europe	*Fagopyrum esculentum*
137	01Z5000020	Orbita	unknown	Unknown	*Fagopyrum esculentum*
148	01Z5000119	SIVA	Slovenia	Europe	*Fagopyrum esculentum*
153	01Z5000038	PI 481639	Bhutan	Asia	*Fagopyrum esculentum*
154	01Z5000039	PI 481640	Bhutan	Asia	*Fagopyrum esculentum*
155	01Z5000043	PI 482595	Zimbabwe	Africa	*Fagopyrum esculentum*
156	01Z5000045	PI 482597	Zimbabwe	Africa	*Fagopyrum esculentum*
160	01Z5000041	PI 481642	Bhutan	Asia	*Fagopyrum esculentum*
172	01Z5000021	Slavyachka	Ukraine	Europe	*Fagopyrum esculentum*
173	01Z5000068	Viktoriya	Ukraine	Europe	*Fagopyrum esculentum*
174	01Z5000100	GREEN COROLLA 2	unknown	Unknown	*Fagopyrum esculentum*
175	01Z5000122	Darina	Slovenia	Europe	*Fagopyrum esculentum*
176	01Z5000144	CD 7273	unknown	Unknown	*Fagopyrum esculentum*
181	UC0101496	RADEKHIVS'KA POLIPSHENA	Ukraine	Europe	*Fagopyrum esculentum*
183	UC0101679		Canada	America	*Fagopyrum esculentum*
192	UC0101979	Malinka	Ukraine	Europe	*Fagopyrum esculentum*
194	UC0100115	BOGATyR	Russian Federation	Eurasia	*Fagopyrum esculentum*

201	UC0100276	Bol'shevik-Albaniya	Albania	Europe	*Fagopyrum esculentum*
202	UC0100277		Nedherlands	Europe	*Fagopyrum esculentum*
206	UC0100282		France	Europe	*Fagopyrum esculentum*
218	UC0100949	Olmemil'	People's republic of Korea	Asia	*Fagopyrum esculentum*
219	UC0101095	Shinano1	Japan	Asia	*Fagopyrum esculentum*
222	UC0100991	Bilorus'ka odnostebel'na	Belarus	Europe	*Fagopyrum esculentum*
225	UC0101109	Gorets' vuz'kolistii	Russian Federation	Eurasia	*Fagopyrum esculentum*
228	UC0102185	Shortandinskaya2	Kazachstan	Eurasia	*Fagopyrum esculentum*
229	UC0100348	Zelenokvitkova 90	Ukraine	Europe	*Fagopyrum esculentum*
230	UC01002208	Arno	Canada	America	*Fagopyrum esculentum*
235	UC0102114	Marta	Belarus	Europe	*Fagopyrum esculentum*
237	UC0102201	Aresa	Belarus	Europe	*Fagopyrum esculentum*
238	UC0102203	Ametist	Belarus	Europe	*Fagopyrum esculentum*
240	64	Nostran	unknown	Unknown	*Fagopyrum esculentum*
244	01Z5000128	Panda	Russian Federation	Eurasia	*Fagopyrum esculentum*
245	01Z5000095	Kora	unknown	Unknown	*Fagopyrum esculentum*
246	01Z5000065	Hruszowska	Poland	Europe	*Fagopyrum esculentum*
7		Bamby	Austria	Europe	*Fagopyrum esculentum*
16	01Z5000055	Arihira Zairai	Japan	Asia	*Fagopyrum esculentum*
35	01Z5000134	Rubra	Russian Federation	Eurasia	*Fagopyrum esculentum*
188	UC0101927		Georgia	Eurasia	*Fagopyrum esculentum*
193	UC0101987	KARMEN	Belarus	Europe	*Fagopyrum esculentum*
79	PI 647612	Clfa 38	United States	America	*Fagopyrum tataricum*
84	PI 199769	PA 160	United States	America	*Fagopyrum tataricum*
134	01Z5100011	903016	Pakistan	Asia	*Fagopyrum tataricum*
140	01Z5100005	PI 481656	Bhutan	Asia	*Fagopyrum tataricum*

Table S2. Descriptive statistics of the 37 qualitative traits in common buckwheat (*F. esculentum*) and Tartary buckwheat (*F. tataricum*).

Trait	Abbreviation	Class	*F. esculentum* (n=190) Frequency	H'	*F. tataricum* (n=51) Frequency	H'	Fisher's Exact
Cotyledon anthocyanin coloration	COC	Absent or very weak	66	0.986	44	0.456	4.04E-10
		Medium	20		1		
		Strong	2		0		
		Weak	102		6		
Cotyledon/ seedling leaf colour	CSCLC	Green	66	0.7	44	0.400	4.51E-11
		Pink	122		7		
		Red	2		0		
Growth and branch shoot habit	GBSH	Erect longer	53	1.240	22	1.127	0.021
		Erect shorter	88		18		
		Semi-erect longer	26		10		
		Semi-erect shorter	23		1		
Leaf number	LNUM	1-3 leaves	78	0.785	9	1.028	0.000
		8-12 leaves	107		18		
		13-15 leaves	5		24		
Time to maturity	MAT	Early	1	0.658	0	0.097	1.32E-06
		Late	128		50		
		Medium	61		1		
Lodging susceptibility	LSUS	Intermediate	105	0.998	20	0.938	4.81E-04
		Very susceptible	42		5		
		Very resistant	43		26		
Total number of flower clusters	TNFC	Few	22	0.867	0	0.466	8.01E-03
		Many	44		9		
		Medium	124		42		
Stem anthocyanin coloration	SAC	Absent or weak	1	0.696	22	1.248	< 2.2E-16
		Medium	73		14		
		Strong	116		4		
		Absent or weak/Medium	0		11		
Nodes in stem	SNN	Many	174	0.289	42	0.466	0.070
		Medium	16		9		
Stem diameter	SDIA	Large	9	0.725	2	0.652	0.875
		Medium	136		39		
		Small	45		10		
Stem colour	STCOL	Green	0	0.924	15	1.286	< 2.2E-16
		Green/Pink	7		11		
		Green/Pink/Red	3		0		
		Green/Red	92		20		
		Pink	1		0		
		Red	87		5		
Intensity of green colour in leaf blade	LBGC	Dark	144	0.707	6	1.039	< 2.2E-16
		Light/Medium	2		2		
		Medium	36		30		
		Medium/Dark	8		13		
Leaf margin colour	LMCOL	Green	190	0.000	18	1.585	< 2.2E-16

		Green/Pink	0		1		
		Green/Red	0		11		
		Pink	0		6		
		Pink/Red	0		6		
		Red	0		9		
Leaf vein colour	LVCOL	Green	14	1.170	3	1.011	0.870
		Green/Pink	88		24		
		Green/Pink/Red	9		2		
		Green/Red	73		22		
		Red	6		0		
Petiole colour	PCOL	Green/Pink	80	1.032	34	0.944	< 2.2E-16
		Green/Pink/Red	27		10		
		Green/Red	82		2		
		Red	1		0		
		Green	0		5		
Leaf blade shape	LBS	Cordate	3	1.607	0	1.209	< 2.2E-16
		Hastate	28		20		
		Hastate/Cordate	42		2		
		Hastate/Sagittate	62		15		
		Sagittate	33		14		
		Sagittate/Cordate	24		0		
Inflorescence: anthocyanin coloration of bud	IAC	Absent or very weak	48	1.105	18	1.253	0.163
		Medium	25		8		
		Strong	11		5		
		Weak	106		20		
Branched inflorescence	BINF	No	6	0.14	7	0.400	7.87E-03
		Yes	184		44		
Colour of inflorescence stalk	CINFS	Green	136	0.597	11	1.191	< 2.2E-16
		Green/Pink	54		10		
		Green/Red	0		1		
		Pink/Red	0		2		
		Red	0		27		
Flower colour	FCOL	Greenish-yellow	1	0.567	20	0.756	< 2.2E-16
		Pink	2		0		
		Pink/Red	1		0		
		White	159		0		
		White/Greenish-yellow	25		0		
		White/Pink	2		0		
		Greenish-yellow/Pink	0		30		
		Greenish-yellow/Red	0		1		
Flower morphology	FMOR	Stamens as long as style	7	0.811	51	0.000	< 2.2E-16
		Stamens longer style	107		0		
		Stamens shorter than style	76		0		
Compactness of inflorescence	CINF	Cyme semi-compact	70	1.436	0	0.000	< 2.2E-16
		Cyme semi-compact/Cyme compact	50		0		
		Cyme compact	16		0		
		Cyme loose	12		51		

		Cyme loose/Cyme semi-compact	42		0		
Seed: shape	SSH	Elliptic	2	0.117	33	0.808	< 2.2E-16
		Ovate	2		15		
		Trullate	186		3		
Seed surface	SS	Irregular	1	0.033	51	0.000	< 2.2E-16
		Smooth	189		0		
Seed colour	SCOL	Brown	8	1.136	46	0.370	5.00E-04
		Brown/Black	3		1		
		Brown/Black/Mottled	3		0		
		Brown/Mottled	136		0		
		Grey	5		0		
		Grey/Brown	2		4		
		Grey/Brown/Mottled	12		0		
		Grey/Mottled	7		0		
		Mottled	14		0		
	Mean			*0.773*		*0.749*	

H', Shannon-Weaver diversity index.

Table S3. List of the 24 SSR markers used for genetic diversity analysis of common buckwheat (*F. esculentum*) and Tartary buckwheat (*F. tataricum*).

SSR marker	M13 label	Forward sequence	Reverse sequence	Tm (touch-down conditions/decreased T per cycle)	Species of origin
Fem1303	FAM	AGGAGACGGGAGAGAAGCAG	GGATGTTTGGGTGATTTCAG	from 60°C to 49.5°C for 0.7 °C	*F. esculentum*
Fem1322	NED	AAGCATTCATTCATTCATTC	GAGTTTGTTGTGTTTGGAGG	from 60°C to 49.5°C for 0.7 °C	*F. esculentum*
Fem1407	HEX	GTGATGAGTAGTTGCCTCTG	CTTGGCTTAGACCTCTCGTA	from 60°C to 49.5°C for 0.7 °C	*F. esculentum*
Fem1582	FAM	TCTGGAGAAGCTAAACCCAC	CCGCAGTTTGTAGGGAGGGA	from 62 °C to 51.5°C for 0.7 °C	*F. esculentum*
Fem1840	NED	ACGACGAAGACAAATGAGGA	ATATGGACGGCCTGGATTAT	from 60°C to 49.5°C for 0.7 °C	*F. esculentum*
SXAU048	HEX	TCCTAGCCTCTTAGGTTAAGG	TTCTAACCGATGTCAAACCAC	from 62 °C to 51.5°C for 0.7 °C	*F. tataricum*
SXAU060	FAM	TCCCAATAGCCAATAGTACATG	GACCTAATTAACCGTTAGCACA	from 60°C to 49.5°C for 0.7 °C	*F. tataricum*
SXAU089	NED	CAAAAGAAAAGTGCCGAAGT	TTATGTCACCGCCATTGTT	from 60°C to 49.5°C for 0.7 °C	*F. tataricum*
SXAU129	HEX	CTCAAAGGATGCCATTGTAAC	GACTTTGAGAACGCCTTGAC	from 60°C to 49.5°C for 0.7 °C	*F. tataricum*
SXAU138	FAM	CACCTGCTACAATACTCTCA	GCTTAATCAACAGTAGGCAC	from 60°C to 49.5°C for 0.7 °C	*F. tataricum*
Ft1_114	NED	CAACAGCATCTTTCCCTTCA	CCATAAACACAGCAACAGCC	from 62 °C to 51.5°C for 0.7 °C	*F. tataricum*
Ft2_1743	HEX	ACCACTGACAATAAGGGGGA	CAAAAGGTTGATGTGGATGG	from 60°C to 49.5°C for 0.7 °C	*F. tataricum*
Ft3_572	FAM	CATCACCCCTCTCAAGACCT	AGAATCCTACCCCGTCCTTT	from 60°C to 49.5°C for 0.7 °C	*F. tataricum*
Ft4_2725	NED	TAGCGATTTGAAGGGGACTT	CGTAACAATGGTCGTTACTCG	from 60°C to 49.5°C for 0.7 °C	*F. tataricum*
Ft5_2899	HEX	AAGCTTCCTTCCATGACCAC	GTTTCTTGTGTGGACCGTTG	from 60°C to 49.5°C for 0.7 °C	*F. tataricum*

Ft6_2849	FAM	ACAATTCATCAAGCGACTCC	CTTTGCCGAATGTAGGGAAT	from 60°C to 49.5°C for 0.7 °C	*F. tataricum*
Ft7_382	NED	TGGTCTCTAAAACGGACCGA	TCGGAACCGGATTCTCTTAC	from 60°C to 49.5°C for 0.7 °C	*F. tataricum*
Ft8_605	HEX	AACGAGGGTACTAACCGGAA	CCCCAGCTGTAAAACAATCA	from 60°C to 49.5°C for 0.7 °C	*F. tataricum*
GB-FE-025	FAM	CAGATGTTACCCGAGGCA	ACCCATATGTCACGAGCG	from 62 °C to 51.5°C for 0.7 °C	*F. esculentum*
GB-FE-121	NED	TCCATACCAAGCAGGTGG	GTGCCTGATGAGGTTCCA	from 60°C to 49.5°C for 0.7 °C	*F. esculentum*
GB-FE-035	HEX	TGCAATGACTTGGAGGAGA	ACCACCATTCAACAAGCG	from 60°C to 49.5°C for 0.7 °C	*F. esculentum*
GB-FE-054	FAM	TGTTGGACTTCCTAGACCTG	CATGAAAAGGGGATGCAA	from 62 °C to 51.5°C for 0.7 °C	*F. esculentum*
TBP5	NED	GGGGATTGATCGAGAAAG	CCGAAGAGTAACTAGGAC	from 60°C to 49.5°C for 0.7 °C	*F. tataricum*
TBP6	HEX	CGGCTAATAAGTCGTTTC	GGATCATAGGTCGTGAAT	from 60°C to 49.5°C for 0.7 °C	*F. tataricum*

Table S4. Correlation matrices of the 37 agro-morphological traits based on Spearman's coefficient in *F. esculentum* (above the diagonal) and *F. tataricum* (below the diagonal). Only significant correlations are shown ($p < 0.01$).

	COC	CSCLC	GBSH	LNUM	MAT	LSUS	TNFC	SAC	SNN	SDIA	STCOL	LBGC	LMCOL	LVCOL	PCOL	LBS	IAC	BINF	CINFS	FCOL	FMOR	CINF	SSH	SS	SCOL	SL	SW	TSW	PH	PL	LBL	LBW	DF	LC	NFCC	NCP	GER
**COC**	1.00	0.88	0.06	-0.28	0.05	-0.04		0.04	0.02	0.21				0.05	0.11	0.05	0.05	-0.06	0.06	0.07	-0.06	0.09	0.12	-0.07	-0.09	0.07	-0.04	-0.08	-0.02	-0.07	-0.02	-0.07		-0.16	-0.25	-0.07	-0.04
**CSCLC**	0.98	1.00	0.11	-0.24	0.05	-0.01	0.02	0.02	0.01	0.19				0.09	0.11	0.03	0.07	-0.06	0.12	0.11	-0.05	0.09	0.14	-0.05	-0.14	0.09		-0.02		-0.09	-0.04	-0.07	0.02	-0.12	-0.21	-0.03	-0.05
**GBSH**	-0.05	0.03	1.00	0.04	-0.02	-0.05	0.04	-0.12	0.09			-0.05		-0.02	-0.03	0.12	-0.03	-0.08	-0.15	-0.05	0.04	-0.15	0.06		-0.12	0.07	0.16	0.14	-0.16	-0.05	-0.08	-0.05	0.11	-0.06	-0.04	-0.06	0.05
**LNUM**	-0.18	-0.18	-0.04	1.00	-0.38	-0.01	-0.02	-0.08	-0.13	-0.18		-0.06		0.08	-0.03	0.15	-0.09	-0.03	-0.10	0.12	0.06	-0.03	-0.12	0.09		0.02	0.09	0.06	0.12	0.18	0.05	0.15	0.12	0.07	0.16	0.06	-0.04
**MAT**	-0.06	-0.06	0.03	-0.24	1.00		-0.02	0.02	-0.08	0.10	-0.04	0.19		-0.06	-0.16	-0.07	-0.01	-0.07	0.17	-0.07	-0.04	0.01		-0.10	-0.04	-0.06	-0.04	-0.05		-0.21	-0.11	-0.17	-0.35	-0.13	-0.08	-0.09	-0.08
**LSUS**	-0.08	-0.11	-0.25	0.15	0.13	1.00	-0.05	0.07	0.03	-0.02	0.14	-0.03		0.08		-0.06	0.13	0.04	0.09	0.04		0.19	0.06	0.06	0.12	-0.02	-0.04		0.03		-0.01	0.01	-0.07	0.02	0.07		0.05
**TNFC**	-0.29	-0.26	0.01	-0.03	0.07	-0.32	1.00	-0.13	-0.02			0.07		0.01	0.02	0.06	-0.03	-0.12	0.00	0.04	0.01	-0.08	0.07	-0.05	0.03	-0.13	-0.10	-0.13	0.06	-0.03	-0.06	-0.02		-0.04	-0.04	0.05	-0.08
**SAC**	0.09	0.11	-0.10	0.03		-0.10	-0.05	1.00	0.01		0.61	-0.14		0.20	0.29		0.07	-0.08	0.12	-0.08	0.12	0.02	-0.06	-0.06	0.01	0.04	-0.05	0.03	0.10	-0.09	-0.09	-0.13	-0.09	-0.05	0.02	0.07	-0.10
**SNN**	-0.18	-0.18	0.61	0.03	-0.07	-0.17	0.21	-0.25	1.00	0.15	-0.05			-0.07	0.05	0.06	0.02	-0.16	-0.07	0.06	0.18	-0.06	0.04	0.02	0.08	-0.15	-0.09	-0.10	-0.53	-0.15	-0.28	-0.31	0.09	-0.19	-0.26	0.28	0.36
**SDIA**	-0.13	-0.14	0.50	-0.27	-0.05	-0.22	0.16	-0.04	0.63	1.00	-0.02	0.11		-0.14	0.05	-0.08	0.01	0.13	0.11	0.12	-0.02	0.09	0.15	0.03	-0.07		-0.06	-0.11	-0.17	-0.11	-0.03	-0.20	-0.09	-0.14	-0.18	-0.05	0.13
**STCOL**	0.10	0.11	-0.10	-0.07	0.10		-0.22	0.66	-0.24	-0.10	1.00	-0.24		0.18	0.39		0.10	-0.03	0.12	-0.02	0.07	-0.07	-0.06	0.05	0.06	0.06	-0.02		0.09	-0.10	-0.08	-0.07	0.01	-0.08	0.06	-0.03	-0.14
**LBGC**	-0.40	-0.38	0.02			-0.04	0.11	-0.24	0.01	0.06	-0.06	1.00		-0.24	-0.34	0.02	-0.04	0.10	0.16	-0.03	0.01	-0.09	0.03	0.04	-0.06	0.05	0.02		-0.04	-0.15	-0.07	-0.16	-0.16	-0.10	-0.09	-0.03	0.09
**LMCOL**	0.16	0.15	-0.02	0.04	0.06	0.04	-0.10	0.24	0.02	-0.07	0.14	-0.09	1.00																								
**LVCOL**	0.37	0.38	-0.01	-0.07	0.16	-0.06	-0.17	0.28	-0.13	0.01	0.08	-0.24	0.17	1.00	0.20		0.08	-0.08	0.09	0.04	0.03	0.10	-0.02	-0.01	0.04	-0.05	-0.01	-0.04	0.13	-0.01		0.08	-0.17	0.01	0.02	0.05	-0.20
**PCOL**	0.31	0.33	0.25	-0.07	-0.04	-0.13	-0.19	0.39	0.03	0.04	0.41	-0.24	0.30	0.30	1.00	0.10	0.08	-0.12	0.05	0.01	0.01	-0.02	0.05	-0.08	0.07	0.03	0.04	0.07	-0.07	0.06	0.03	0.06	0.02	-0.03	-0.07		-0.13
**LBS**	-0.12	-0.14	0.09	0.18	0.17	0.20	0.13	0.06	0.12	-0.02		0.38	0.39	0.13	0.05	1.00	-0.02	-0.12		-0.02	0.07	-0.02	0.11	-0.07	0.05	0.17	0.25	0.25	0.02	0.05	-0.06	-0.02	0.04	-0.04	-0.03		-0.03
**IAC**	0.21	0.23	-0.43	0.05	-0.16	0.17	-0.32	0.13	-0.42	-0.30	0.31	0.18	-0.03	0.17	-0.06	0.00	1.00	0.04	0.15	0.06	-0.02	0.19	-0.08	-0.06	0.03	0.09	0.04	0.10	0.04	-0.11	-0.17	-0.16	-0.03	0.02	0.05	0.05	0.08
**BINF**	0.16	0.16	-0.58	-0.14	0.06	0.23	-0.18	0.28	-0.71	-0.36	0.23		-0.15	0.05	-0.24	-0.17	0.46	1.00	0.05	0.06	-0.04	0.26	-0.03	0.40	-0.03	-0.03	-0.18	-0.13	0.19	0.11	0.17	0.09	0.03	0.42	0.43	-0.15	-0.09

**CINFS**	-0.02	-0.04	-0.52	-0.16	0.12	0.23	-0.14	0.07	-0.48	-0.32	0.28	0.28	0.16	-0.14	-0.23	0.16	0.40	0.50	**1.00**	-0.05	-0.03	0.17	0.09	0.05	-0.15	0.06	-0.08	-0.11	0.10	-0.13	-0.04	-0.08	-0.15	-0.16	-0.02	0.04	
**FCOL**	0.11	0.07	-0.58	-0.19	-0.17	0.25	-0.13	0.33	-0.46	-0.25	0.44	-0.02	0.16	0.07	0.14	0.05	0.49	0.48	0.50	**1.00**	0.04	0.05	0.04	0.02	-0.08	-0.01	-0.02	-0.04	0.05	-0.08	0.02	0.05		0.05	0.07	0.04	-0.06
**FMOR**																					**1.00**	-0.03	0.09	-0.08	0.10	-0.02	0.08	0.04		-0.02	-0.03	-0.08	-0.02	0.05	0.02	0.04	0.03
**CINF**																							-0.02	0.10	0.00	0.03	-0.06	0.02	0.28	-0.04	0.16	0.13	-0.12	0.18	0.25	-0.01	
**SSH**	0.23	0.20	0.40	0.06	-0.10	-0.16	-0.03	0.06	0.37	0.26	0.03	-0.28	0.05	-0.05	0.37	0.12	-0.25	-0.39	-0.47	-0.20			**1.00**	-0.01	0.02	-0.02	0.06	0.01	-0.09	-0.03	-0.03	-0.03		-0.06	-0.01	0.02	-0.03
**SS**																								**1.00**	0.02	0.00	-0.07	-0.12		0.05	0.05	0.06	-0.03	0.02	0.09	-0.07	0.02
**SCOL**	0.31	0.29	0.16	0.02	-0.05	-0.08	-0.23	0.42	-0.15	-0.11	0.26	-0.20	0.35	0.08	0.46	0.14	-0.07	-0.08	-0.17	0.09			0.55		**1.00**	-0.06	-0.09	0.09	-0.04	0.15	0.05		0.06		-0.07	0.05	-0.10
**SL**	-0.32	-0.30		0.37	-0.39	-0.20	0.16		0.01	-0.11	-0.02	0.40	0.02	-0.19		0.10	-0.02	-0.13	0.05	-0.13			-0.22		-0.03	**1.00**	0.73	0.62	0.09	-0.08	-0.13	-0.09	0.13	-0.09	-0.11	-0.14	0.10
**SW**	0.06	0.05	0.27	0.44	-0.56	-0.27	0.01	-0.10	0.30	0.07	-0.20	0.06	0.11	-0.11	0.30	0.11	-0.14	-0.43	-0.36	-0.22			0.37		0.28	0.63	**1.00**	0.82	-0.02	-0.10	-0.23	-0.08		-0.11	-0.14	-0.19	0.10
**TSW**			-0.09	0.40	-0.34	-0.14	0.04	-0.05	-0.06	-0.18	-0.08	0.29	0.14	-0.20	0.10	0.14	0.04		0.09	-0.05			0.02		0.11	0.77	0.70	**1.00**	0.02	-0.04	-0.17	-0.06		-0.09	-0.13	-0.18	-0.02
**PH**	0.06	0.06	-0.23	-0.03	0.11	0.41	-0.39		-0.53	-0.25	-0.04	-0.03	-0.05	-0.16	-0.08	-0.11	0.07	0.43	0.37	0.19			-0.12		0.05	-0.28	-0.29	-0.06	**1.00**	0.16	0.28	0.29	-0.13	0.38	0.38	-0.09	-0.29
**PL**	0.11	0.10	-0.34		0.16	0.18	0.03	-0.08	-0.45	-0.52	-0.04	0.08	-0.19	-0.24	-0.08	0.02	0.05	0.28	0.10	0.20			-0.05		0.10	-0.03	-0.04	0.11	0.27	**1.00**	0.59	0.59		0.08	-0.02	-0.05	-0.21
**LBL**	-0.13	-0.12	-0.20	0.03	0.03	0.07	0.08	-0.06	-0.17	-0.19	-0.11	0.07	0.04	-0.28	-0.17		-0.02	0.19	-0.05	-0.08			-0.14		-0.10	0.10	-0.07	0.11	0.17	0.44	**1.00**	0.83		0.17	0.16	-0.06	-0.19
**LBW**	0.25	0.27	-0.27	0.04	0.08	0.34	-0.30	0.18	-0.48	-0.46	0.20	0.10	-0.07	0.16	0.03	0.13	0.43	0.49	0.47	0.36			-0.18		0.07		-0.16	0.18	0.27	0.14	-0.26	**1.00**	0.03	0.19	0.18	-0.07	-0.20
**DF**	0.20	0.21	-0.21		-0.13		0.16	0.19	-0.23	-0.22	0.18	-0.42	-0.28	0.09	-0.13	-0.35	0.16	0.33	-0.26	0.16					-0.06	-0.18	-0.18	-0.17	-0.15	0.25	0.18	0.04	**1.00**	0.14	0.06	0.21	0.11
**LC**	0.14	0.20	-0.32	0.05	-0.08	0.11	-0.09	0.10	-0.38	-0.33	0.09	-0.11	-0.20	0.03	-0.16	-0.28	0.28	0.53	0.23	0.29			-0.28		-0.26	-0.06	-0.27	0.01	0.37	0.22	0.22	0.37	0.44	**1.00**	0.73	0.06	-0.12
**NFCC**	0.06	0.10	-0.48	0.05	-0.04	0.04	-0.02	0.34	-0.41	-0.36	0.26	-0.08	-0.13	0.07	-0.19	-0.23	0.34	0.69	0.36	0.41			-0.41		-0.29	0.03	-0.34	0.10	0.17	0.15	0.18	0.40	0.49	0.79	**1.00**	0.08	-0.11
**NCP**	-0.10	-0.10	0.47	0.02	-0.18	-0.28	0.27	-0.30	0.68	0.38	-0.15	0.10	-0.12	-0.19	0.15	0.04	-0.22	-0.56	-0.38	-0.21			0.37		-0.14	0.04	0.40	0.05	-0.43	-0.18	-0.16	-0.30	-0.10	-0.29	-0.32	**1.00**	0.19
**GER**	0.02		-0.19	-0.18	0.46	0.25	-0.05	-0.11	-0.24	-0.18	0.03	0.27	-0.10	0.11	-0.14	0.17	0.10	0.21	0.35	0.09			-0.11		-0.03		-0.20	0.05	0.07	0.35	0.07	0.38	-0.15	0.05	0.06	-0.22	**1.00**

Table S5. Marker-trait associations for common buckwheat (*F. esculentum*) generated by general linear model (GLM) and mixed linear model (MLM) at $p < 0.05$.

Trait	Marker	GLM		MLM	
		p	R^2 (%)	p	R^2 (%)
Branched inflorescence	Ft3_572	0.001	34.23	0.028	19.70
	Ft4_2725	0.014	19.39	0.001	30.29
	Ft5_2899	1.40E-05	31.10	–	–
	Ft8_605	0.001	20.38	0.005	19.27
	SXAU138	–	–	0.011	33.54
Compactness of inflorescence	Ft2_1743	0.007	15.55	–	–
Colour of inflorescence stalk	Fem1407	0.036	26.44	–	–
	Ft8_605	0.022	13.35	–	–
	SXAU089	0.050	43.18	–	–
Cotyledon: anthocyanin coloration	Ft3_572	–	–	0.026	19.80
	Ft4_2725	0.006	20.61	–	–
Cotyledon/seedling leaf colour	Ft4_2725	0.045	17.19	–	–
	TBP_5	0.046	13.21	–	–
Days to flowering	Fem1840	0.042	27.19	–	–
Flower colour	Fem1322	–	–	0.009	37.81
	Fem1407	0	37.54	–	–
	Fem1528	–	–	0.009	39.32
	Fem1840	0	40.40	–	–
Growth and branch shoot habit	TBP_5	0.013	14.93	–	–
	TBP_6	0.033	7.78	–	–
Germination period	Ft5_2899	0.050	18.93	–	–
Inflorescence: anthocyanin coloration of bud	Fem1840	0.025	30.49	–	–
	GB_FE_054	0.013	24.81	–	–
	GB_FE_121	–	–	0.040	25.85
Leaf blade: intensity of green colour	Ft4_2725	0.047	16.79	–	–
	Ft8_605	0.006	26.88	–	–
	SXAU060	0.041	13.84	–	–
Leaf blade length	Ft5_2899	0.001	23.69	–	–
	SXAU048	0.002	42.18	–	–
	SXAU060	0.028	14.03	–	–
Leaf blade shape	Ft8_605	0.037	23.43	–	–
Leaf blade width	Fem1528	0.001	43.01	–	–
	Ft3_572	0.016	27.37	–	–
	Ft4_2725	0.018	18.04	–	–
	Ft5_2899	0.001	25.29	–	–
	SXAU048	0.014	38.95	–	–
	TBP6	0.013	8.85	–	–
Length of cyme	Fem1407	0.040	29.34	–	–
Leaf number	Ft1_114	0.012	26.62	–	–
	SXAU060	0.040	14.09	–	–
Number of cymes per plant	Fem1840	0.011	32.24	–	–
	Ft4_2725	–	–	0.039	20.88
	Ft5_2899	0.004	22.39	–	–
Number of flowers clusters per cyme	Fem1407	0.033	30.21	–	–

	Ft4_2725	–	–	0.023	22.41
	Ft5_2899	0.003	23.96	–	–
	Ft8_605	0.023	24.89	–	–
	SXAU129	0.020	36.10	–	–
Plant height	Ft1_114	0.021	22.29	–	–
Petiole length	Fem1528	0.007	41.42	–	–
	Ft5_2899	0.020	20.30	–	–
	SXAU048	0.040	37.76	–	–
	SXAU060	0.008	16.55	–	–
Seed colour	Fem1322	0.013	31.24	–	–
	Ft1_114	–	–	0.041	15.52
	Ft2_1743	0.024	15.33	–	–
	Ft3_572	–	–	0.037	18.66
	Ft4_2725	0.015	18.67	–	–
	SXAU048	0.006	41.28	–	–
	SXAU138	0.022	21.84	–	–
Stem: diameter	Ft1_114	0.029	24.96	–	–
	Ft8_605	0.002	18.97	0.010	17.97
Seed length	Fem1407	–	–	0.024	44.60
	Fem1528	0	47.59	–	–
	Fem1840	0.019	31.99	–	–
	Ft1_114	0.023	25.09	–	–
	Ft3_572	–	–	0.017	20.17
	Ft4_2725	0.012	19.30	–	–
	Ft7_382	0.002	29.68	–	–
	GB_FE_035	–	–	0.023	21.72
	GB_FE_121	0.001	25.93	–	–
	TBP_5	–	–	0.049	7.49
	TBP_6	0.024	8.31	–	–
Stem: number of nodes	Ft1_114	0.014	25.07	–	–
	Ft3_572	5.34E-08	42.82	–	–
	GB_FE_054	–	–	0.008	17.55
	SXAU138	–	–	3.68E-04	42.87
	TBP_5	0	20.30	0.004	11.49
	TBP_6	1.81E-04	13.68	–	–
Stem: anthocyanin coloration	Ft1_114	0.003	28.19	–	–
	SXAU129	–	–	0.026	27.77
Stem colour	Ft1_114	0.002	28.93	–	–
	GB_FE_025	–	–	0.029	10.14
	GB_FE_035	0.014	10.39	–	–
	SXAU129	–	–	0.013	29.67
Seed width	Fem1303	0.035	16.70	–	–
	Ft3_572	–	–	0.036	18.82
	Ft4_2725	0.003	21.21	–	–
	TBP_6	0.027	8.11	–	–
Plant: total number of flower clusters	Fem1528	0.010	40.54	–	–
1000-seed weight	Fem1407	–	–	0.044	43.45
	Fem1528	0.004	43.12	–	–
	TBP_6	0.036	7.91	–	–

GLM, general linear model; MLM, linear mixed model; R^2, proportion of phenotypic variance explained by SSR markers.

Table S6. Marker-trait associations for Tartary buckwheat (*F. tataricum*) generated by general linear model (GLM) and mixed linear model (MLM) at $p < 0.05$.

**Trait**	**Marker**	**GLM**		**MLM**	
		p	R^2 (%)	p	R^2 (%)
Branched inflorescence	Fem1322	0.002	33.12	–	–
	Fem1407	–	–	0.020	63.94
	Ft1_114	0.035	8.09	0.028	48.58
	Ft2_1743	0.022	13.77	–	–
	Ft4_2725	0.001	44.02	–	–
	Ft5_2899	0.004	26.31	–	–
	GB_FE_054	0.009	22.54	–	–
	GB_FE_121	0.027	21.46	–	–
	SXAU129	0.011	25.17	–	–
Colour of inflorescence stalk	Ft4_2725	0.011	45.98	–	–
	Ft6_2849	0.026	26.70	–	–
	GB_FE_025	0.050	22.81	–	–
	GB_FE_121	0.026	24.87	–	–
	SXAU048	0.002	13.29	–	–
	SXAU060	0.002	24.04	–	–
	SXAU089	–	–	0.037	25.03
	TBP_5	0.004	19.97	–	–
Cotyledon: anthocyanin coloration	Fem1840	0.042	24.62	–	–
	Ft7_382	0.025	33.52	–	–
Cotyledon/seedling leaf colour	Fem1840	0.009	31.75	–	–
	Ft3_572	0.045	30.41	–	–
	Ft7_382	0.004	42.15	–	–
	SXAU089	0.043	17.87	–	–
Days to flowering	Fem1528	0.026	53.60	–	–
	Ft6_2849	7.03E-06	61.21	–	–
	Ft7_382	–	–	0.033	46.96
	GB_FE_025	0.032	33.75	–	–
Germination period	Fem1303	0.017	14.68	–	–
	Fem1322	–	–	0.028	14.68
	SXAU138	0.041	64.57	–	–
Inflorescence: anthocyanin coloration of bud	Fem1840	4.03E-04	36.72	–	–
	Ft5_2899	0.020	29.90	–	–
	SXAU048	0.016	9.41	–	–
Leaf blade length	Ft6_2849	0.029	36.29	–	–
Leaf blade shape	SXAU060	0.009	27.46	–	–
	SXAU089	0.003	28.88	–	–
	SXAU129	–	–	0.027	23.86
Length of cyme	Fem1407	0.007	38.01	–	–
	Ft4_2725	0.002	55.99	–	–
	Ft6_2849	0.008	33.56	–	–
	Ft7_382	0.037	28.54	–	–
Leaf margin colour	Ft5_2899	0.031	31.38	–	–
	GB_FE_035	0.001	22.23	–	–

	GB_FE_054	0.030	29.13	–	–
	GB_FE_121	0.028	31.77	–	–
	GB_FE_121	–	–	0.017	16.60
Leaf number	SXAU089	0.008	21.33	–	–
Lodging susceptibility	Fem1840	0.008	27.72	–	–
	Ft2_1743	0.009	20.50	–	–
	Ft4_2725	0.039	48.27	–	–
	Ft5_2899	0.004	34.11	–	–
	GB_FE_054	0.009	29.83	–	–
	SXAU048	0.001	15.33	–	–
	SXAU060	0.021	20.25	–	–
	TBP_5	0.007	21.26	–	–
Leaf vein colour	Ft3_572	0.002	45.61	–	–
	Ft4_2725	0.029	62.73	–	–
	Ft5_2899	0.049	33.00	–	–
	Ft6_2849	0.002	48.48	–	–
	Ft7_382	0.018	40.27	–	–
	SXAU048	0.018	11.29	–	–
	SXAU060	0.023	25.36	–	–
Number of cymes per plant	Ft2_1743	1.50E-04	29.21	–	–
	Ft5_2899	1.26E-04	39.84	–	–
	GB_FE_054	1.92E-04	37.11	–	–
	SXAU048	0.029	7.13	–	–
	TBP_5	0.004	21.05	–	–
Number of flowers clusters per cyme	Fem1322	0.001	36.54	–	–
	Fem1407	0.040	26.62	–	–
	Fem1407	–	–	0.039	49.07
	Ft5_2899	0.014	24.87	–	–
	Ft6_2849	0.001	32.30	–	–
	Ft7_382	–	–	0.038	39.58
	Ft8_605	0.050	14.50	–	–
	SXAU138	0.044	45.47	–	–
	TBP6	–	–	0.022	22.61
Petiole colour	Fem1407	0.043	30.19	–	–
	Fem1528	0.019	45.62	–	–
	SXAU048	0.008	10.22	–	–
	TBP_5	0.045	14.27	–	–
Plant height	Fem1840	0.016	29.98	–	–
	Ft2_1743	0.029	19.74	–	–
	Ft1_114	–	–	0.004	67.43
Seed colour	Ft2_1743	1.38E-05	39.72	–	–
	Ft5_2899	0.002	42.36	–	–
	Ft6_2849	–	–	0.013	51.89
	Ft7_382	0.049	32.33	–	–
	Ft8_605	–	–	0.010	58.44
	SXAU129	1.02E-05	57.09	–	–
Stem diameter	GB_FE_025	0.041	22.31	–	–
Seed length	Fem1840	0.016	30.96	–	–
	Ft3_572	2.28E-04	49.82	–	–

	Ft4_2725	0.036	58.53	0.029	40.09
	Ft5_2899	0.033	33.05	–	–
	Ft7_382	0.002	46.16	–	–
	GB_FE_054	0.002	41.55	–	–
	TBP_5	0.001	30.81	–	–
Stem: number of nodes	Fem1322	0.012	21.53	–	–
	Fem1407	0.048	17.72	–	–
	Fem1840	0.017	14.17	–	–
	Ft2_1743	9.35E-12	32.04	–	–
	Ft3_572	0.002	20.05	–	–
	Ft4_2725	0.033	27.24	–	–
	Ft5_2899	1.03E-04	24.00	–	–
	Ft6_2849	8.02E-06	27.71	–	–
	Ft7_382	0.001	23.06	0.021	51.72
	GB_FE_054	0.008	16.64	–	–
	SXAU060	0.003	14.32	–	–
	SXAU138	–	–	0	60.20
Stem: anthocyanin coloration	GB_FE_035	0.014	14.78	–	–
Seed shape	Fem1528	0.019	30.58	–	–
	Fem1840	0.005	19.59	0.048	74.08
	Ft2_1743	–	–	0.031	15.01
	Ft5_2899	2.86E-04	27.09	–	–
	GB_FE_025	0.013	20.26	–	–
	GB_FE_035	–	–	0.013	49.62
	SXAU048	0.010	6.96	0.048	31.55
	SXAU060	0.009	15.16	–	–
Seed width	Fem1322	0.025	42.32	–	–
	Fem1407	0.041	38.03	–	–
	Ft4_2725	6.57E-05	73.18	–	–
	GB_FE_054	2.24E-08	64.39	–	–
	TBP_5	4.66E-07	49.77	–	–
Plant: total number of flower clusters	Fem1840	0.011	33.93	–	–
	Ft4_2725	0.045	60.03	–	–
	Ft7_382	0.029	37.54	–	–
1000-seed weight	Fem1322	–	–	0.020	17.73
	Fem1407	0.006	45.93	–	–
	Fem1840	0.033	27.57	–	–
	Ft3_572	0.002	43.00	–	–
	Ft4_2725	2.66E-04	71.63	–	–
	Ft5_2899	0.013	36.29	–	–
	Ft7_382	0.003	43.72	–	–
	GB_FE_054	0.010	34.98	–	–
	TBP_5	0.004	27.27	–	–

GLM, general linear model; MLM, linear mixed model; R^2, proportion of phenotypic variance explained by SSR markers.
